# Regeneration in starved planarians depends on TRiC/CCT subunits modulating the unfolded protein response

**DOI:** 10.15252/embr.202152905

**Published:** 2021-06-30

**Authors:** Óscar Gutiérrez‐Gutiérrez, Daniel A Felix, Alessandra Salvetti, Elias M Amro, Anne Thems, Stefan Pietsch, Andreas Koeberle, K Lenhard Rudolph, Cristina González‐Estévez

**Affiliations:** ^1^ Leibniz Institute on Aging‐Fritz Lipmann Institute (FLI) Jena Germany; ^2^ Department of Clinical and Experimental Medicine Unit of Experimental Biology and Genetics University of Pisa Pisa Italy; ^3^ Chair of Pharmaceutical/Medicinal Chemistry Institute of Pharmacy Friedrich‐Schiller‐University Jena Jena Germany; ^4^ Michael Popp Institute and Center for Molecular Biosciences Innsbruck (CMBI) University of Innsbruck Innsbruck Austria

**Keywords:** chaperonin, ER stress, hematopoietic stem cell, planarian, starvation, Metabolism, Regenerative Medicine

## Abstract

Planarians are able to stand long periods of starvation by maintaining adult stem cell pools and regenerative capacity. The molecular pathways that are needed for the maintenance of regeneration during starvation are not known. Here, we show that down‐regulation of chaperonin TRiC/CCT subunits abrogates the regeneration capacity of planarians during starvation, but TRiC/CCT subunits are dispensable for regeneration in fed planarians. Under starvation, they are required to maintain mitotic fidelity and for blastema formation. We show that TRiC subunits modulate the unfolded protein response (UPR) and are required to maintain ATP levels in starved planarians. Regenerative defects in starved CCT‐depleted planarians can be rescued by either chemical induction of mild endoplasmic reticulum stress, which leads to induction of the UPR, or by the supplementation of fatty acids. Together, these results indicate that CCT‐dependent UPR induction promotes regeneration of planarians under food restriction.

## Graphical abstract

It is unknown how starved planarians maintain their ability to regenerate. This study shows that CCT‐dependent UPR induction promotes planarian regeneration specifically under food restriction.

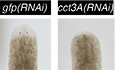

## Introduction

Regeneration is widespread in the animal kingdom with almost every phylum having species with the capacity to regrow some parts of their bodies. Planarians are an extreme example of regeneration. They possess a large population of stem cells, approximately 15–25% of the total cell number in the parenchyma, that permits planarians to fully regenerate their bodies in a few days (Baguñà, 1976b). Planarian stem cells (pluripotent and specialized) are the only proliferative cells and are able to collectively give rise to any planarian cell type (Baguñà, 1976b; Reddien, 2018). During the process of regeneration, planarians need to cope with a massive demand for new cells to form the regenerative blastema by inducing hyper‐proliferation of their stem cells (Reddien, 2018). Indeed, regeneration is a highly energy‐demanding process that requires the animals to allocate resources (Maginnis, 2006). Remarkably, planarians are able to stand starvation maintaining the relative number of stem cells and the regenerative power just like fed or growing planarians (González‐Estévez *et al*, 2012a; Felix *et al*, 2019). Stem cell maintenance during starvation serves as a strategy to allow for a rapid growth when a more favourable nutritional environment is encountered or for being primed for a regenerative response to injury (Felix *et al*, 2019). However, it is currently unknown how starved planarians cope metabolically with regeneration and/or what genetic programmes are necessary for the maintenance of regeneration specifically under conditions of starvation.

Proteome integrity is essential for the functionality of cells and the survival of organisms. The maintenance of protein homeostasis (proteostasis) is regulated by a complex network, which coordinates protein synthesis, folding, trafficking, aggregation, disaggregation and degradation of proteins. This balanced network is constantly affected by many factors including mutations and age (Balch *et al*, 2008; Hipp *et al*, 2019). Interestingly, interventions that increase lifespan such as dietary restriction (DR) or reduction of insulin/insulin‐like growth factor 1 (IGF‐1) signalling are associated with enhanced mechanisms regulating proteostasis (Cohen *et al*, 2009).

Chaperones are a component of the proteostasis network and are responsible for assisting the *de novo* folding and protection of existing proteins from proteotoxic stress (Labbadia & Morimoto, 2015). There are specific chaperones for each of the protein‐folding compartments in a cell. The stress response at the cytosol induces the expression of chaperones, co‐chaperones and chaperonins under different sorts of stress. Chaperones can act alone or in combination with various co‐chaperones to regulate client‐substrate interactions, folding, disaggregation, degradation and trafficking within the cell. Compared to other chaperones, the HSP60/chaperonin member TRiC (TCP1‐ring complex or chaperonin containing TCP1, also known as CCT) recognizes a smaller repertoire of substrates and is necessary for folding about 5‐10% of newly synthesized proteins including actin and tubulin (Saibil, 2013). It also binds to misfolded proteins regulating their aggregation. Indeed, it has been predicted that late folding intermediates or misfolded species are preferred substrate conformers of the TRiC (Horwich *et al*, 2007). The endoplasmic reticulum (ER) is the major organelle for lipid synthesis and the biosynthesis/folding and maturation of proteins and another component of the proteostasis network. When overloaded with misfolded proteins, the unfolded protein response (UPR) is activated at the ER leading to decreases in global protein translation and specific transcription of stress response genes that promote proteostasis and cell survival. Misfolded, aggregated or damaged proteins are degraded through the proteasome or autophagy. If the UPR fails to restore the ER to normality, ER stress can promote apoptosis (Labbadia & Morimoto, 2015).

Here we perform transcriptional profiling of stem cells at different nutritional states to unravel regulators of regeneration during starvation. We found that down‐regulation of TRiC subunits impedes planarian regeneration only during starvation by down‐regulating the UPR. The study reveals that CCTs are necessary for mitotic fidelity and the maintenance of genome integrity of planarian stem cells during starvation. We also find that CCTs regulate ATP levels during starvation. Indeed, supplementation of fatty acids or chemical induction of mild ER stress is sufficient to rescue impairments in survival and regeneration of CCT‐depleted planarians exposed to food starvation. We validate that CCT depletion abrogates the UPR activation in mouse hematopoietic stem and progenitor cells (HSPCs) under glucose deprivation, a mammalian regenerative system. Our work identifies CCT‐mediated UPR induction as a mechanism that contributes to the unique capacity of planarians to be able to fully regenerate even under starved conditions.

## Results

### Transcriptional profiling reveals TRiC subunits as potential regulators of regeneration depending on nutritional states

**Figure EV1:**
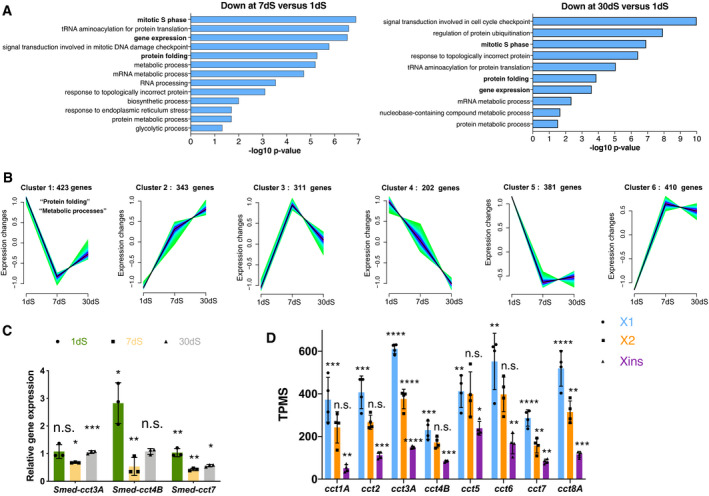
Transcriptional profile of stem cells at different nutritional states finds TRiC subunits enriched in stem cells and differentially regulated at 1dS and 30dS versus 7dS AGO enrichment analysis for biological processes of the 814 down‐regulated genes (*q*‐value < 0.1) at 7dS versus 1dS and of the 491 down‐regulated genes (*q*‐value < 0.1) at 30dS versus 1dS in X1 (stem cells). *q*‐value obtained by the Benjamini–Hochberg test. No enriched biological processes were found up‐regulated. For better visualization, similar enriched GO terms based on the same subset of genes were manually removed to reduce redundancy. In bold are the GO terms commented in Results.BClustering generated from 2070 DEGs found in stem cells (X1) according to their expression during different nutritional conditions (1dS, 7dS and 30dS). Number of genes assigned to every cluster is showed on the upper part of every graph. Cluster 1 is characterized by a U shape.CRelative expression of *cct* transcripts at 1, 7 and 30 days of starvation in X1 (stem cells). Error bars are SD from the mean. Asterisks refer to the condition just before and 1dS refers to 30dS and indicate *P* < 0.001 (three asterisks), *P* < 0.01 (two asterisks), *P* < 0.05 (one asterisk) and n.s. indicates not significant using two‐tailed Student's test with equal sample variance. *n* = 3 replicates (5 planarians each) per time point.DExpression levels of the different *cct* genes at 30 days of starvation in TPMs (transcripts per million). X2 corresponds to stem cell progeny (stem cells in G0/G1). These TMPs correspond to the data shown in Fig 1A. Error bars are SD from the mean. Asterisks refer to the condition before and X1 refers to Xins. *P* < 0.0001 (four asterisks), *P* < 0.001 (three asterisks), *P* < 0.01 (two asterisks), *P* < 0.05 (one asterisk) and n.s. indicates not significant using two‐tailed Student's test with equal sample variance. *n* = 4 replicates (40 planarians per replicate). Source data are available online for this figure.

In order to investigate the transcriptional profiles of stem cells in different nutritional states, we sorted stem cells by FACS (X1 subfraction: S and G2/M cell cycle phase stem cells) (Hayashi *et al*, 2006) from 1, 7 and 30 days starved planarians (1dS, 7dS and 30dS, respectively) and performed RNA‐seq and pairwise comparison between the different nutrient conditions. By performing gene ontology (GO) enrichment, we found that “mitotic S phase” was among the most overrepresented biological processes down‐regulated in X1 stem cells at 7dS and 30dS versus 1dS (Fig EV1A, Dataset EV1a‐c), agreeing with 1dS planarians being under a mitotic response to feeding (Baguñà, 1976a). We observed that most of the components of the translation machinery were also down‐regulated in response to starvation (Fig EV1A and Dataset EV1d) standing in line with the down‐regulation of translation in response to nutrient deprivation in other organisms (Hansen *et al*, 2007).

**Figure 1:**
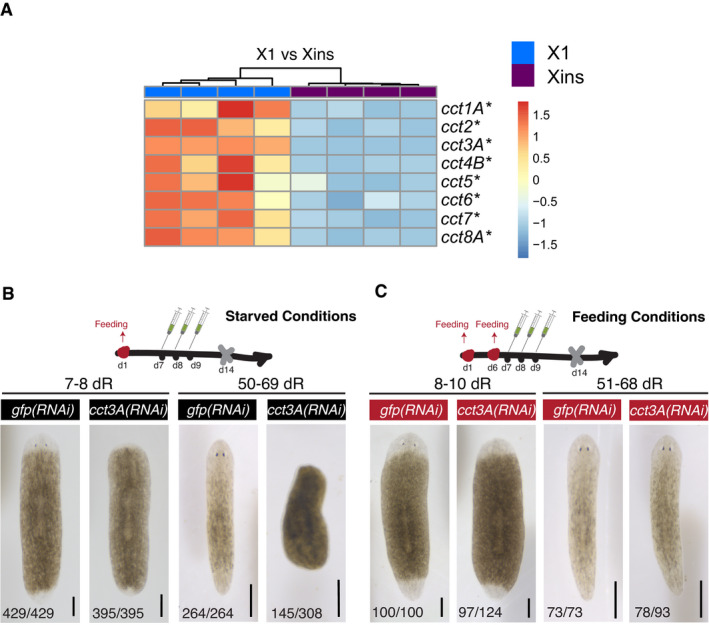
The expression of *cct3A* is enriched in stem cells and necessary for blastema formation during starvation AThe expression heat map shows that all the somatic *cct* subunits from TRiC are up‐regulated in X1 (stem cells) compared to Xins (differentiated cells) at 30dS. The colour‐coded scale indicates Row Z‐score of normalized TPMs (transcripts per million) values per replicate. The asterisks indicate that these genes are significant differentially expressed (*q*‐value (FDR) < 0.01).BRNAi injections schedule in starved conditions. Planarians are at 14dS when the amputation is performed (indicated by the grey cross at day 14). Live images show that *cct3A(RNAi)* planarians form a minimal blastema compared to controls at the time points shown. At the bottom are the number of planarians with the phenotype shown. The remaining planarians are dead by the time point of regeneration shown. dR indicates days of regeneration.CRNAi injections schedule in feeding conditions. One extra feeding in respect to (B) is introduced 1 day prior to injections. Planarians are at 8dS when the amputation is performed. The live images show that most of the *cct3A(RNAi)* planarians regenerate like controls. At the bottom are the number of planarians with the phenotype shown. At 8‐10dR, the remaining planarians are either dead (4/124) or have a tiny blastema (23/124) and either died or regenerated later. At 51‐68dR, the remaining planarians are either dead (10/93) or have a tiny blastema and died later (5/93). Data information: Scale bars, 300 µm. Source data are available online for this figure.

Interestingly, the term “protein folding” (Fig EV1A) was one of the most up‐regulated gene categories during the feeding response (1dS) when compared to 7dS and 30dS with many of the transcripts corresponding to cytosolic chaperones (25/41 up in 1dS versus 7dS and 12/21 up in 1dS versus 30dS) (Dataset EV1e, f). In addition to their functions in folding of *de novo* synthesized proteins, many chaperones are also induced under conditions of environmental stress and are involved in protein refolding, disaggregation, trafficking and degradation also in humans (Yang *et al*, 2016). Grouping differentially expressed genes (DEGs) according to expression profile trajectories into clusters (Kumar & Futschik, 2007) identified one U shaped cluster with a relative expression decrease at 7dS versus 1dS but a recovery of gene expression to feeding conditions (1dS) at 30dS (cluster 1 in Fig EV1B and Dataset EV2a‐b). Of note, most of the transcripts related to "protein folding" in this cluster (23/27) were enriched in X1 (stem cells) when compared to Xins (differentiated cells) (Dataset EV2c‐d) including most of the subunits of the chaperonin TRiC (6/8) (Figs 1A and EV1C and D and Dataset EV2d). A search for other components of TRiC (not included in cluster 1) corroborated that they were also enriched in stem cells and followed a trend of down‐regulation at 7dS but a recovery at 30dS compared to 1dS (Fig 1A, Dataset EV2d). In agreement with our observation, it has been suggested that high levels of CCT subunits are hallmarks of hESCs (Noormohammadi *et al*, 2016). Based on these data, we wondered whether TRiC subunits could be potential regulators of regeneration in certain nutritional states.

### TRiC subunits are necessary for blastema formation specifically in starved planarians

The TRiC is formed by a double‐ring complex of 8 units codified by different genes (*cct1* to *cct8*) that belong to the chaperone family of HSP60 (Kubota *et al*, 1995). Homologs of each *cct* gene and some gonad‐specific paralogs have been previously identified in the sexual strain of *S. mediterranea* (Counts *et al*, 2017; Rouhana *et al*, 2017). Since the sexual‐specific *ccts* had almost undetectable expression levels in our RNA‐seq data sets of the asexual strain, we focused on the somatic *ccts*.

**Figure EV2:**
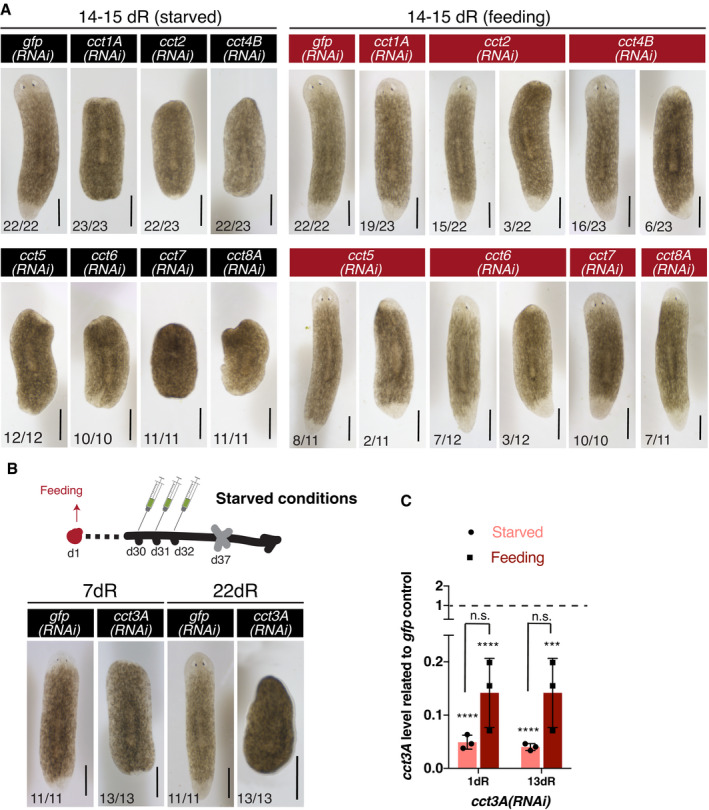
TRiC subunits are necessary for blastema formation specifically in starved planarians ALive images show that RNAi for any *cct* gene in starved conditions (14dS when the amputation is performed) leads to minimal blastema formation compared to controls at the time point shown whereas in feeding conditions (one extra feeding is introduced 1 day prior to injections) results in most planarians regenerating as controls. At the bottom are the number of planarians with the phenotype shown. The remaining planarians are dead by the time point of regeneration shown.BRNAi injections schedule in starved conditions (37 days of starvation when the amputation is performed which is indicated by the grey cross at day 37). Live images show that *cct3A(RNAi)* planarians form a minimal blastema when compared to controls at the time points shown. At the bottom are the number of planarians with the phenotype shown.CExpression of *cct3A* in *cct3A(RNAi)* planarians related to *gfp* RNAi injected either under starved (14dS) or feeding conditions at 1 and 13 days of regeneration. Error bars are SD from the mean. *P* < 0.0001 (four asterisks), *P* < 0.001 (three asterisks) and n.s. indicates not significant using two‐tailed Student's test with equal sample variance. *n* = 3 replicates (5 planarians each) per time point. Data information: dR, days of regeneration. Scale bars, 500 µm. Source data are available online for this figure.

Aiming to understand the function of *ccts* during regeneration of starved planarians, we designed RNAi schedules under different nutritional states. We injected planarians with either dsRNA targeting *gfp* as control or any of the *ccts* for three consecutive days and then amputated heads and tails 5 days later to follow the regeneration of the trunks. To analyse regeneration under starvation, planarians were either 14 days (hereafter referred to as "starved conditions") or 37 days starved when performing the amputation (Figs 1B and EV2A and B). The down‐regulation of each one of the 8 *ccts* during the starvation schedule resulted in defective blastema (new regenerating tissue) formation and death of the animals starting at 30 days after amputation (Figs 1B and EV2A and B). To analyse regeneration under feeding, one extra feeding was allocated 1 day before the first injection (hereafter referred to as "feeding conditions"). The down‐regulation of each of the 8 *ccts* in feeding conditions did not affect regeneration as most of the amputated planarians formed a normal blastema (Figs 1C and EV2A). Importantly, the level of RNAi down‐regulation was not altered by the feeding (Fig EV2C) and feeding did also not rescue the previously described phenotypes of *Smed‐smg‐1* or *Smed‐tor* RNAi (González‐Estévez *et al*, 2012b) (Appendix Fig S1A and B). Together these results showed that the feeding protocol did not interfere with the efficiency of the RNAi.

A previous report on starved planarians which were fed multiple times with dsRNA for *ccts* showed a rapid demise of the planarians (Counts *et al*, 2017). Although they performed dsRNA by feeding instead of injection and used sexual instead of asexual planarians, we tried to design a similar schedule adapted to our specific conditions. Starved planarians were fed after RNAi injections, and we observed that there was also a rapid failure (see for *cct3A* RNAi in Appendix Fig S1C). However, when planarians that were under the feeding condition were fed after RNAi injections, we observed that they regenerated even better (Appendix Fig S1D). Altogether indicates that the metabolic condition ("starved" or "feeding") before down‐regulation of the genes determines the phenotype: no regeneration in case of "starved" and regeneration in case of "feeding".

We conclude that TRiC subunits are required for blastema formation and successful regeneration in starved planarians but dispensable in fed planarians.

### Down‐regulation of *cct3A* in starved planarians leads to mitotic failure during regeneration

**Figure 2:**
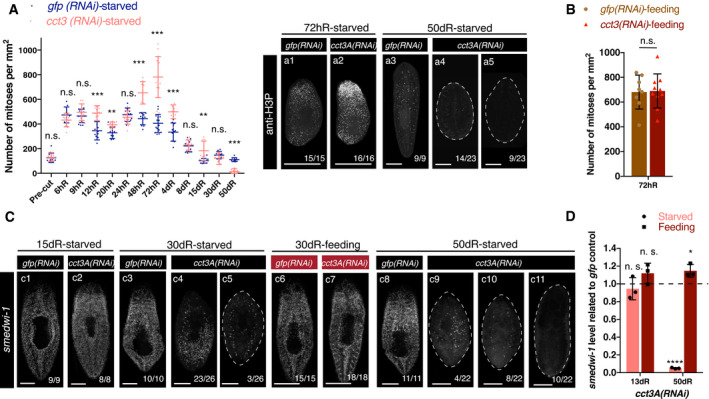
*cct3A* RNAi in starved conditions shows increased numbers of mitoses after both mitotic peaks and stem cell depletion at late time points of regeneration AThe graph shows the mitotic numbers of trunks during different time points of regeneration in the starved condition for *cct3A* RNAi and controls. Error bars are SD from the mean and asterisks indicate *P* < 0.001 (three asterisks), *P* < 0.01 (two asterisks) and n.s. indicates not significant using two‐tailed Student’s test with equal variance. *n* ≥ 9 planarians per time point. Also shown are maximum projections of representative trunks labelled with anti‐H3P at different time points of regeneration in the starved condition. On the bottom, the number of planarians with the phenotype shown from the total is displayed. Note that at 50dR planarians show few (a4) or no mitoses (a5). The dashed line delimits the body of the planarian.BThe graph shows the mitotic numbers during 72 h of regeneration in feeding conditions for *cct3A* RNAi and controls. Error bars are SD from the mean, and n.s. indicates not significant using two‐tailed Student's test with equal sample variance; *n* ≥ 8 planarian per time point.CMaximum projections of representative trunks after FISH for *smedwi‐1* at different time points of regeneration under starved and feeding conditions. On the bottom, the number of planarians with the phenotype shown from the total is displayed. At 15dR, the expression levels are similar. At 30dR, some planarians have almost no expression (c5) and at 50dR almost all planarians show no expression (c10 and c11) in starved conditions. Under the feeding condition the expression levels at 30dS are similar. On the bottom, the number of planarians with the phenotype shown from the total is displayed. The dashed line delimits the body of the planarian.DRelative expression of *smedwi‐1* at 13 and 50 days of regeneration after either *cct3A* or *gfp* RNAi under feeding and starved conditions. Error bars are SD from the mean and asterisks indicate *P* < 0.001 (three asterisks), *P* < 0.05 (one asterisk), and n.s. indicates not significant using two‐tailed Student’s test with equal variance. *n* = 3 replicates (5 planarians each) per time point. Data information: hR, hours of regeneration; dR, days of regeneration. Scale bars, 1 mm (a1‐a2) and 250 µm (a3‐a5, c1‐c3 and c6‐c9) and 500 µm (c4‐c5). Source data are available online for this figure.

The observation that *ccts* are necessary for blastema formation suggested that they may regulate stem cell proliferation and/or differentiation. We therefore examined the pattern of mitoses during regeneration in starved planarians by using a Histone H3 phosphorylated at serine 10 antibody (anti‐H3P) (Hendzel *et al*, 1997). We chose one of the *ccts* with the highest phenotype penetrance (*Smed‐cct3A*) as representative to further investigate TRiC subunits. It has been shown that planarian amputation triggers two mitotic peaks early in regeneration that contribute to blastema formation and growth (Baguñà, 1976b; Wenemoser & Reddien, 2010). We observed that *cct3A(RNAi)* animals had an increased number of H3P^+^ stem cells just after both mitotic peaks when compared to control planarians (Fig 2A). Remarkably, these defects did not occur in feeding conditions (Fig 2B). At 15dR *cct3A(RNAi),* animals also showed a slight increase of mitoses, whereas after 50dR mitotic activity was nearly abolished when compared to controls (Fig 2A).

**Figure EV3:**
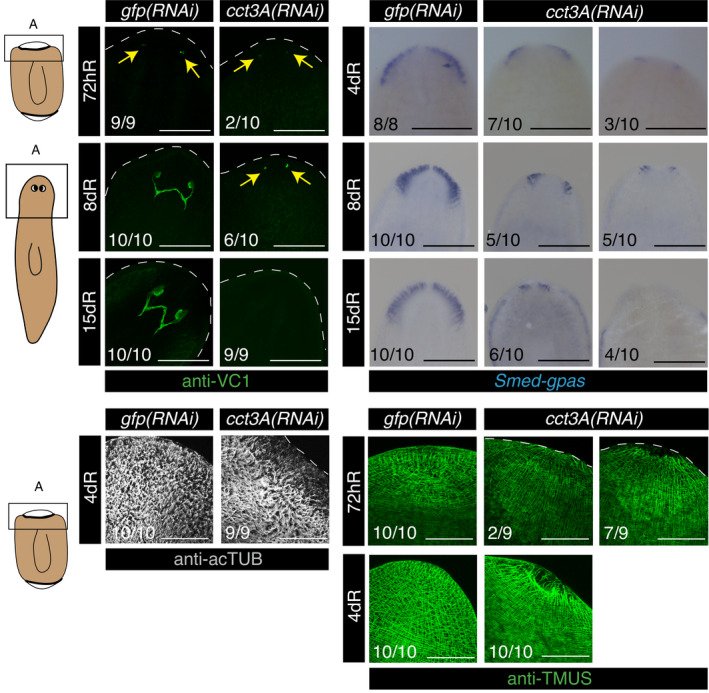
*cct3A* RNAi show minimal differentiation during regeneration in starved conditions The squares at the cartoons indicate the regions shown in the panels. The images show minimal differentiation of eyes (anti‐VC1), brain (*Smed‐gpas*), epidermal cilia (anti‐acTUB) and muscle (anti‐TMUS) in anterior wounds of *cct3A* RNAi compared to controls. At the bottom are the number of planarians with the phenotype shown. Arrows indicate the differentiating eyes. Scale bars, 300 µm (VC1 images), 500 µm (*gpas* images), 150 µm (TMUS and AC‐TUB images). hR indicates hours of regeneration, and dR indicates days of regeneration. Source data are available online for this figure.

Next, we sought to determine whether *cct3A* RNAi affects stem cell numbers by conducting fluorescent *in situ* hybridization (FISH) and qPCR for *smedwi‐1*, a marker that labels proliferating stem cells in planarians (Reddien *et al*, 2005). At 30dR stem cell loss was evident in some planarians, and by 50dR, most of the planarians showed few if any stem cells (Fig 2C and D). In contrast, *cct3A* RNAi did not affect stem cell numbers under feeding conditions (Fig 2C and D), fitting with the maintenance of regenerative capacity observed under this condition (Fig 1C). In agreement with the lack of blastema growth in *cct3A(RNAi)* planarians in starved conditions, we observed minimal differentiation of eyes (anti‐VC1) (Sakai *et al*, 2000), brain (*Smed‐gpas*) (Iglesias *et al*, 2011), epidermal cilia (anti‐acTUB) (Iglesias *et al*, 2011) and muscle (anti‐TMUS) (Cebrià *et al*, 1997) in anterior wounds (Figs 1B and EV3).

**Figure 3:**
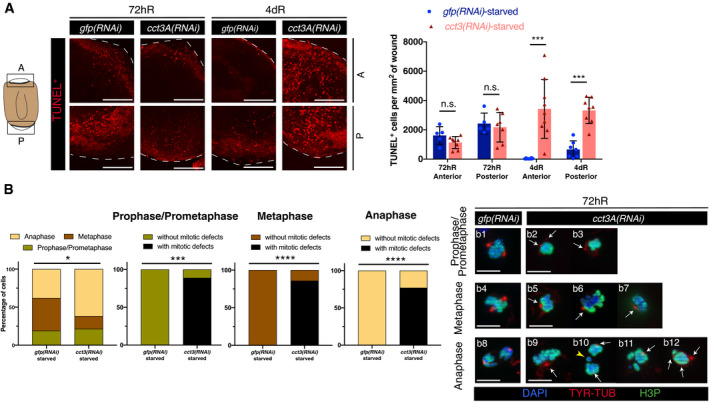
*cct3A* RNAi during starvation leads to mitotic defects, chromosomal aberrations and cell death at wound sites early during regeneration AThe cartoon represents a regenerating trunk and the squares display the region analysed at the anterior “A” and posterior “P” blastemas. The images are TUNEL maximum projections of representative blastemas from 72 h and 4 days regenerating trunks in starved conditions. The dashed line delimits the body of the planarian. The graph shows the number of TUNEL^+^ cells per mm^2^. The differences in cell death are not significant (n.s.) at 72hR (two‐tailed Student's test with equal sample variance) and significant (***P < 0.001 using two‐tailed Student’s test with equal variance) at 4dR. *n* ≥ 5 planarians per condition.BPercentage of stem cells in different mitotic phases and the percentage of defective mitotic figures in 72hR anterior blastemas of *cct3A* RNAi and controls in starved conditions after double immunostaining with anti‐tyrosine‐tubulin and anti‐H3P (****P < 0.0001, ***P < 0.001, *P < 0.05 using two‐sided Chi‐square test; the number of cells analysed is displayed in Appendix Fig S2B); *n* ≥ 5 planarians. Representative images are shown. Nuclei are stained with DAPI. Arrows indicate abnormal organization or number of spindle poles and the yellow arrowhead indicates chromosome lagging. Asymmetrical karyokinesis is observed in b11 and b12. Data information: dR, days of regeneration; hR, hours of regeneration. Scale bars, 4 µm (A), 10 µm (B). Source data are available online for this figure.

The increase in mitotic stem cells in combination with the lack of blastema growth in *cct3A(RNAi)* planarians under starved conditions suggested that *cct3A* is required for mitotic progression under these conditions. Since mitotic arrest can increase cell death, TUNEL staining was conducted on regenerating *cct3A(RNAi)* planarians and controls in starved conditions at the time point which showed the highest accumulation of mitosis and the time point just after (72hR and 4dR). Interestingly, *cct3A* RNAi induced a massive increase in TUNEL^+^ cells in both anterior and posterior wounds (Fig 3A) including stem cells (Appendix Fig S2A) at 4dR. Since the increase in apoptosis occurred only after the mitotic peak and not during the peak itself, we speculated that *cct3A(RNAi)* impairs mitotic fidelity. In line with this assumption, double immunostaining with anti‐tyrosin‐tubulin (de Sousa *et al*, 2018) and anti‐H3P revealed a significant decrease in the percentage of stem cells in metaphase (from ˜43% in controls to ˜17%) and an increase in anaphase (from ˜38% in controls to ˜62%) (Fig 3B, Appendix Fig S2B). In addition, more than 70% of all mitotic figures were defective (Fig 3B, Appendix Fig S2B). Interestingly, in the feeding condition the distribution of the different phases of cell cycle in *cct3A(RNAi)* planarians was similar to controls and we only observed a low number (30%) of defective figures (Appendix Fig S2C–E). This indicates that *cct3A* is required for mitotic fidelity specifically under starvation.

### CCT subunits induce the unfolded protein response (UPR), which is essential for mitotic fidelity during regeneration of starved planarians

**Figure 4:**
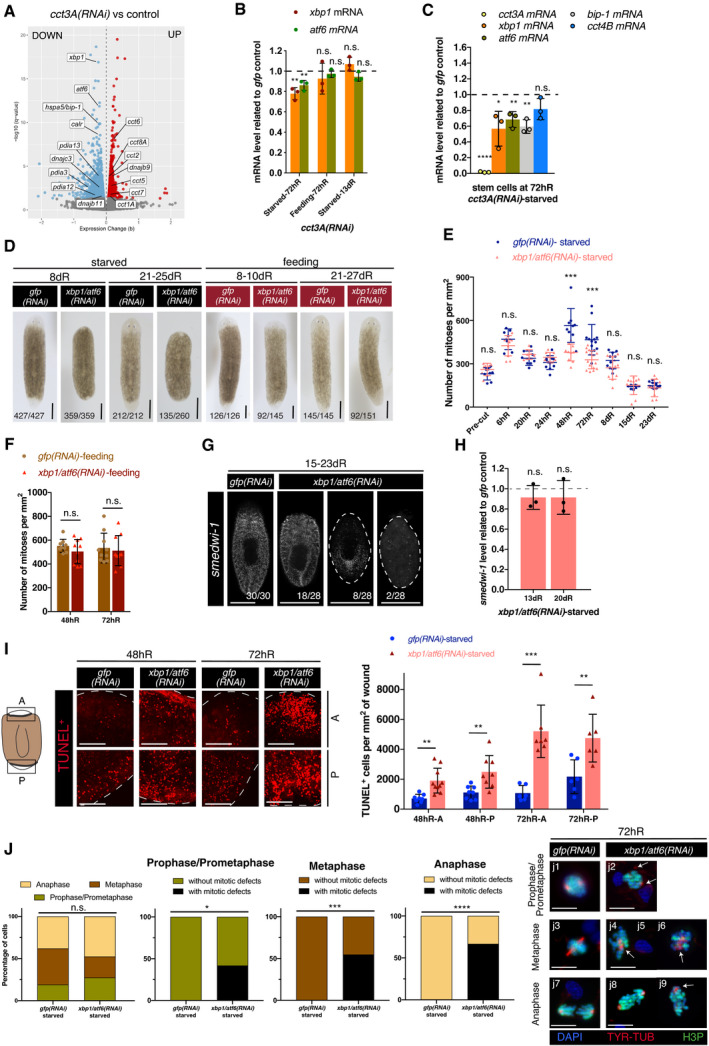
*cct3A* RNAi down‐regulates the UPR components *xbp1 and atf6* whose down‐regulation leads to a similar phenotype as *cct3A* AVolcano plot displaying DEGs in starved *cct3A(RNAi)* planarians compared to controls at 72hR (*q*‐value < 0.05). Y‐axis indicates the negative log10 of the false discovery rate (FDR) (q‐ value). X‐axis indicates the beta values (b), a biased estimator of the fold change. For better visualization, 13 dots with ‐log10 (*q*‐value) > 20 are not displayed. This includes *cct3A* with *q* = 0. Notice that when down‐regulating *cct3A* in starved planarians, *xbp1*, *atf6*, *bip‐1* and a repertoire of ER chaperones are down‐regulated, whereas *dnajb9*, a repressor of the UPR, and other *ccts* are up‐regulated.BRelative expression of *xbp1* and *atf6* related to *gfp* control at 72 h and 13 days of regeneration during either starving or feeding conditions in *cct3A(RNAi)* animals. The graph shows that *cct3A* RNAi down‐regulates *xbp1* and *atf6* specifically during starvation at 72hR. *n* = 3 replicates (5 planarians each) per time point.CThe graph shows that both *cct3A* RNAi down‐regulates *xbp1*, *atf6* and *bip‐1* specifically at 72hR during starvation in X1 stem cells. 50 planarians per replicate (three replicates) were used to obtain the X1 population (stem cells in S and G2/M).DLive images show that *xbp1/atf6(RNAi)* planarians in starved conditions form a minimal blastema when compared to controls while at the feeding condition most of them can regenerate as controls. The remaining planarians in the starved condition are dead by the time point of regeneration shown. In the feeding condition, the remaining planarians at 8‐10dR are either dead (13/145) or have a tiny blastema and died or regenerated later (40/145) while at 21‐27dR are dead.EMitotic numbers during different time points of regeneration in starved conditions for *xbp1/atf6* RNAi and controls. *n* ≥ 7 planarians per time point.FMitotic numbers in feeding conditions for *xbp1/atf6* RNAi and controls. *n* ≥ 9 planarians per time point.GMaximum projections of representative regenerating trunks after FISH for *smedwi‐1* in starved conditions. The dashed line delimits the body of the planarian.HRelative expression of *smedwi‐1* related to controls at 13 and 20 days of regeneration of *xpb1/atf6* RNAi in starved conditions. *n* = 3 replicates (5 planarians each) per time point.ITUNEL maximum projections of representative blastemas from regenerating trunks in starved conditions. The dashed line delimits the body of the planarian. The graph shows the number of TUNEL^+^ cells per mm^2^ at the same time points in anterior (A) and posterior (P) blastemas. *n* ≥ 6 planarians per time point.JQuantification of the percentage of stem cells in different mitotic phases and the percentage of defective mitotic figures in 72hR anterior blastemas of *xbp1/atf6* RNAi and controls in starved conditions after double immunostaining with anti‐tyrosine‐tubulin and anti‐H3P (****P < 0.0001, ***P < 0.001, *P < 0.05, n.s. indicates not significant using two‐sided chi‐square test); *n* ≥ 5 planarians. Representative images are shown. Arrows indicate abnormal organization or number of spindle poles. j8 displays asymmetrical distribution of chromosome content. Data information: In (B, C, E, F, H and I), error bars are SD from the mean and asterisks indicate *P* < 0.0001 (four asterisks), *P* < 0.001 (three asterisks), *P* < 0.01 (two asterisks), *P* < 0.05 (one asterisk) and n.s. indicates not significant using two‐tailed Student’s test with equal variance; at the bottom are the number of planarians with the phenotype shown. dR, days of regeneration; hR, hours of regeneration. Scale bars, 500 µm (D and G), 100 µm (I) and 10 µm (J). Source data are available online for this figure.

**Figure EV4:**
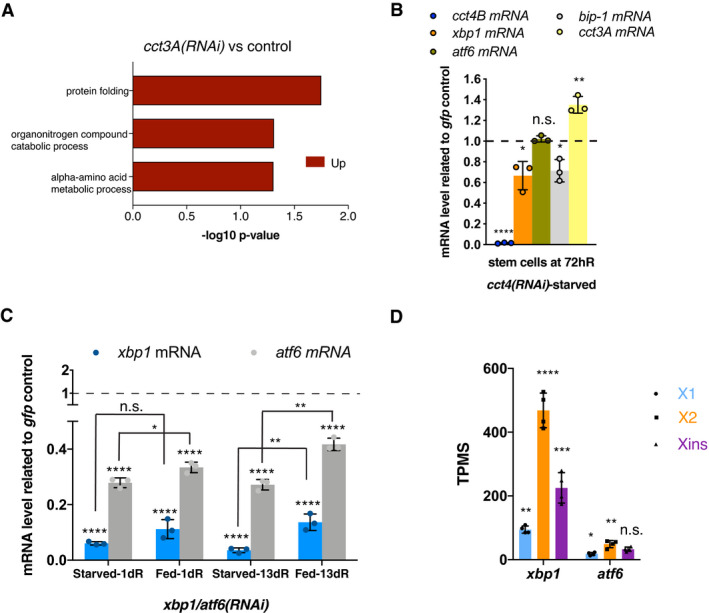
*cct3A* down‐regulates components of the UPR AGO enrichment analysis for biological processes of the 982 up‐regulated genes (*q*‐value < 0.05) in starved *cct3A* RNAi planarians versus controls at 72hR. *q*‐value was obtained with the Benjamini–Hochberg test. No enriched biological processes were found down‐regulated. For better visualization, similar enriched GO terms based on the same subset of genes were manually removed to reduce redundancy.BThe graph shows that *cct4B* RNAi down‐regulates *xbp1* and *bip‐1* and up‐regulates *cct3A* specifically at 72hR during starvation in stem cells. Error bars are SD from the mean. 50 planarians per replicate (3 replicates) were used to obtain the X1 population (stem cells). *P* < 0.0001 (four asterisks), *P* < 0.01 (two asterisks), *P* < 0.05 (one asterisk) and n.s. indicates not significant using two‐tailed Student's test with equal sample variance.CExpression of *xbp1 and atf6* at 1 and 13 days of regeneration after *xbp1/atf6(RNAi)* related to controls during either starving or feeding conditions. The graphs show that *xbp1/atf6* RNAi down‐regulates *xbp1* and *atf6* during starvation and feeding at both time points. Error bars are SD from the mean. Asterisks indicate *P* < 0.0001 (four asterisks), *P* < 0.01 (two asterisks) and *P* < 0.05 (one asterisk), and n.s. indicates not significant using two‐tailed Student's test with equal sample variance. *n* = 3 replicates (5 planarians each) per time point.DExpression levels of *xbp1 and atf6* at 30 days of starvation in the X1, X2 and Xins FACS populations in TPMs (transcripts per million). Error bars are SD from the mean. Asterisks refer to the condition before and X1 refers to Xins and indicate *P* < 0.0001 (four asterisks), *P* < 0.001 (three asterisks), *P* < 0.01 (two asterisks) and *P* < 0.05 (one asterisk), and n.s. indicates not significant using two‐tailed Student's test with equal sample variance. *n* = 4 replicates (40 planarians each) per time point. Source data are available online for this figure.

In order to identify *cct3A*‐dependent pathways that control mitosis under starvation, we performed RNA‐seq on starved *cct3A(RNAi)* planarians and controls at 72hR (Figs 4A and EV4A and Dataset EV3a‐d). Strikingly, among the top down‐regulated genes (with the lowest q‐value) upon *cct3A* RNAi were the components of the unfolded protein response in the endoplasmic reticulum (UPR^ER^) *xbp1* (decrease of 15.44%) and *atf6* (decrease of 13.55%) (Fig 4A). Validation in starved *cct3A(RNAi)* whole planarians by qPCR showed a decrease of 22.15% for *xbp1* and 13.88% for *atf6* (Fig 4B). These two transcription factors are crucial for two of the three main UPR^ER^ branches responsible for the recovery of ER homeostasis or the induction of apoptosis (Labbadia & Morimoto, 2015). We also found down‐regulated a repertoire of ER chaperones, known to be transcriptionally induced by *xbp1* and/or *atf6* (Li & Lee, 2006; Shoulders *et al*, 2013), including *hspa5/bip*, a master regulator of the UPR sensor activation (Li & Lee, 2006; Malhotra & Kaufman, 2007) and a *bona‐fide* marker of the UPR activation when analysed at the mRNA and/or protein level (Kozutsumi *et al*, 1988; Yoshida *et al*, 1998) (Fig 4A). Of note, we also observed the chaperone *dnajb9*, a repressor of the UPR (Amin‐Wetzel *et al*, 2017), to be up‐regulated (Fig 4A). Remarkably, the down‐regulation of *xbp1* and *atf6* was stronger in stem cells with a 43.22% reduction of *xbp1* and 31.58% of *atf6* (Fig 4C) than in whole *cct3A(RNAi)* planarians (Fig 4B) and there was also a strong reduction of *bip* (40.42% reduction, Fig 4C).

Interestingly, we observed that the effect on the UPR was not specific of *cct3A*, as down‐regulating another randomly chosen subunit (*cct4B*) also led to a decrease in *xbp1* and *bip* levels (Fig EV4B).

In order to investigate whether the observed down‐regulation of the UPR^ER^ was causally involved in the mitotic failure and defective blastema of *cct3A(RNAi)* planarians under starved conditions, we performed RNAi experiments for *Smed‐xbp1*, *Smed‐atf6*, *Smed‐bip‐1* and additionally also *Smed‐bip‐2* and *Smed‐bip‐3* (a second and third planarian *bip* not differentially expressed upon *cct3A* RNAi (Fig 4D, Appendix Fig S3A and B and Dataset EV2a). Strikingly, RNAi for *bip‐1* (Appendix Fig S3B) and double RNAi for *xbp1/atf6* (Figs 4D and EV4C) phenocopied the regeneration failure in starved planarians but normal regeneration under feeding conditions as seen in *cct3A(RNAi)* planarians (Fig 1B and C). Interestingly, regenerative failure of *xbp1/atf6(RNAi)* planarians under starved conditions mimicked many of the characteristic features of regeneration failure in *cct3A(RNAi)* animals, such as aberrant mitosis, increased apoptosis during regeneration (including X1 stem cells) and lack of differentiation (Fig 4E–J, Appendix Fig S4A–C). However, no accumulation of mitotic stem cells was evident (Fig 4E) and the depletion of stem cells at later time points of regeneration was not observed in most of the planarians (Fig 4E, G and H). *xbp1/atf6(RNAi)* planarians started to die by 15dR and all the planarians were dead by 35dR (Fig 4D), far faster than *cct3A* RNAi where 47% of the planarians were still alive at 50‐69dR (Fig 1B). Compared to *cct3A* that is enriched in S and G2/M phase stem cells (X1 fraction), *xbp1* and atf6 are more enriched in G0/G1 (X2 fraction) and differentiated cells (Xins) (Fig EV4D). Therefore, it is possible that down‐regulation of *xbp1/atf6* affects not only mitotic stem cells but also differentiating and differentiated cells. This could explain the faster lethality phenotype which prevents the observation of possible long‐term effects in mitotic stem cells such as stem cell depletion in all planarians.

Together, these results indicate that *cct3A*‐dependent *xpb1/atf6* expression contributes to maintain the regenerative capacity of planarians under starved conditions by preventing mitotic failure.

### Treatment with a mild dose of the UPR inducer DTT further supports that TRiC subunits function through the UPR

**Figure EV5:**
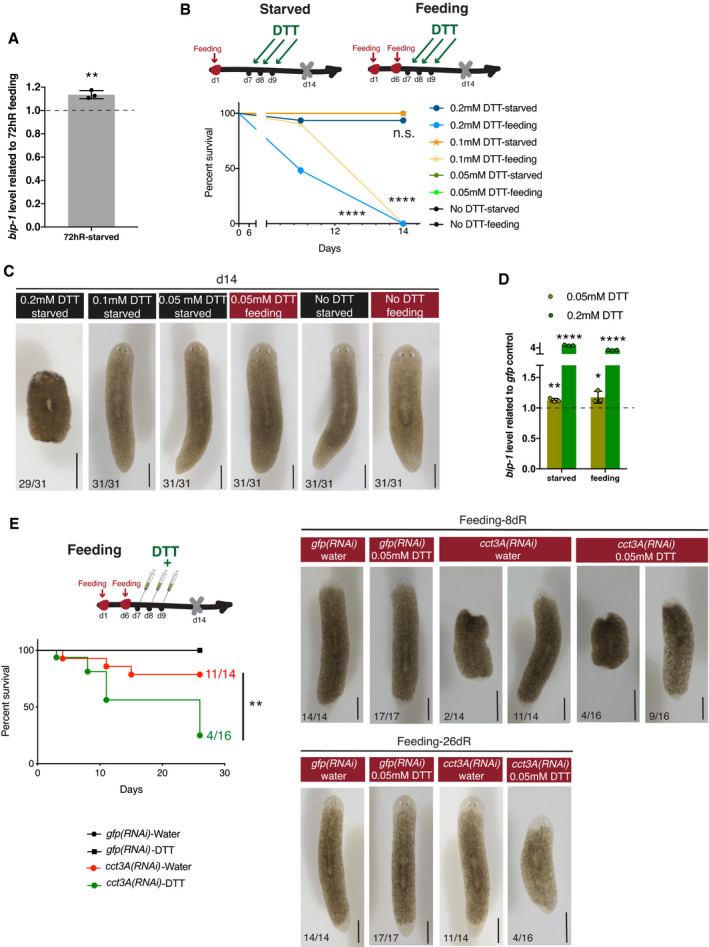
Supplementary experiments on DTT treatments AExpression of *bip‐1* in 72hR planarians in starved conditions related to 72hR planarians in feeding conditions. Error bars are SD from the mean. *P* < 0.01 (two asterisks) using two‐tailed Student's test with equal sample variance. *n* = 3 replicates (5 planarians each).B, CThe Kaplan–Meier curve shows 100% survival of intact planarians treated with 0.05 mM DTT in both starved and feeding conditions. 0.2 mM and 0.1 mM DTT leads to death of all treated animals in feeding conditions by day 14. While 0.2 mM DTT in starved planarians leads to some death, the rest of the planarians showed wounds and lysis by day 14 as displayed in the images. All starved planarians treated with 0.1 mM DTT were normal, and after amputation at day 14, they all regenerated normally. At the concentration of 0.05 mM all planarians, both in the starved and feeding conditions, looked normal after the treatments and regenerated well after the amputation. Four asterisks indicate *P* < 0.0001 of 0.2 mM DTT and 0.1 mM DTT treatments on the feeding conditions respect to controls, and n.s. indicates not significant respect to controls with log‐rank (Mantel–Cox) test. The rest of conditions are not significant. Images are representative surviving animals for the different conditions. At the bottom are the number of planarians with the phenotype shown from a total of 31 planarians per condition in two independent experiments. The rest of planarians are dead at this time point. Scale bars, 1 mm.DExpression of *bip‐1* in planarians in starved and feeding conditions 1 h after the last day of treatment with DTT (0.05 and 0.2 mM). The levels of *bip‐1* increase after exposure to DTT and the increase is bigger with higher doses of DTT. Error bars are SD from the mean. *P* < 0.0001 (four asterisks), *P* < 0.01 (two asterisks) and *P* < 0.05 (one asterisk) using two‐tailed Student's test with equal sample variance. *n* = 3 replicates (5 planarians each) per time point.EThe Kaplan–Meier curve demonstrates decreased survival of *cct3A* RNAi animals when treated with DTT under feeding conditions. Percentages indicate the number of survivals at the indicated time points. Two asterisks indicate *P* < 0.01 with log‐rank (Mantel–Cox) test. Images are representative surviving animals for the different conditions at this time point. At the bottom are the number of planarians with the phenotype shown. The rest of planarians are dead at the displayed time point. dR indicates days of regeneration. Scale bars, 500 µm. Source data are available online for this figure.

**Figure 5:**
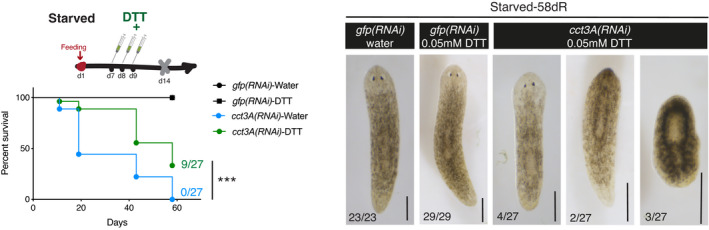
DTT promotes survival in starved *cct3(RNAi)* planarians The Kaplan–Meier curve demonstrates increased survival of *cct3A* RNAi animals when treated with DTT under starved conditions. Percentages indicate the number of survivals at the indicated time points. Three asterisks indicate *P* < 0.001 with log‐rank (Mantel‐Cox) test. Images are representative surviving animals for the different conditions at the displayed time point of regeneration. At the bottom are the number of planarians with the phenotype shown. The rest of planarians are dead at the displayed time point. Data information: dR, days of regeneration. Scale bars, 300 µm. Source data are available online for this figure.

To test whether the UPR down‐regulation contributes to the regenerative failure of *cct3A* RNAi during starvation, we employed the chemical ER‐stressor dithiothreitol (DTT) (Kaufman, 1999). We observed that the expression levels of the ER stress marker *bip‐1* increased during early time points of regeneration in starved control planarians (not RNAi injected) when compared to feeding conditions (Fig EV5A). We reasoned that if the failure in up‐regulating the UPR contributed to the regenerative impairment in *cct3A* RNAi, low dose DTT may have the potential to rescue this deficiency by independently inducing ER stress and the UPR^ER^. First, we determined a low level of ER stress that could induce an ER stress response (*bip‐1* expression) without perturbing regeneration in control planarians under feeding and starved conditions (Fig EV5B–D). Next, we conducted a regeneration experiment with or without adding a mild dose of DTT (0.05mM) for 3.5h prior to RNAi injections (Fig 5). Interestingly, at 58dR we observed that while DTT‐untreated *cct3A(RNAi)* planarians in starved conditions could not regenerate and were all dead (0% survival) as observed before (Fig 1B), 33.33% of DTT‐treated *cct3A(RNAi)* planarians survived with 14.81% completely regenerated (Fig 5).

We also treated planarians with DTT in the feeding condition (Fig EV5E). We observed that most of the DTT‐treated *cct3A(RNAi)* planarians that were alive by 8dR (9/13) showed blastema formation and differentiation. However, DTT‐treated *cct3A(RNAi)* planarians finally survived significantly less (25%) than DTT‐untreated *cct3A(RNAi)* planarians by 26dR (78.6%) (Fig EV5E). This suggests that combining feeding, *cct3A(RNAi)* and DTT treatment has a negative effect in survival without generally affecting early regeneration.

Overall, our results indicate that the UPR down‐regulation contributes to the regenerative failure of *cct3A(RNAi)* planarians during starvation.

### *Cct3*‐knockdown attenuates the UPR^ER^ in freshly isolated mouse hematopoietic stem cells and progenitor cells (HSPCs) in response to glucose deprivation

**Figure 6:**
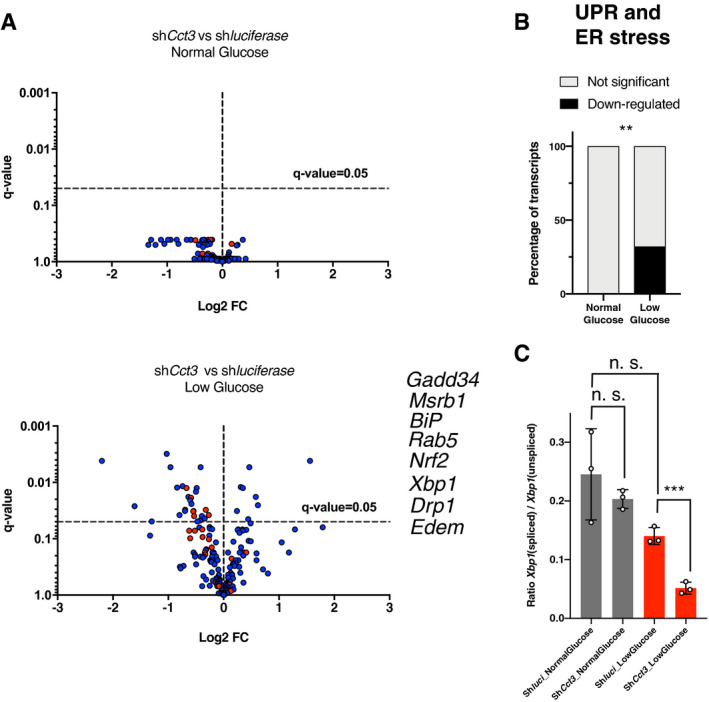
NanoString analysis of stress response genes on normal and low glucose cultured mouse LSK cells (hematopoietic stem cells and multipotent hematopoietic progenitors) AVolcano plots displaying shRNA‐*Cct3*‐ and shRNA‐*luciferase*‐infected LSK cells cultured either in normal glucose (upper graph) or low glucose conditions (lower graph). Dots represent all the NanoString analysed transcripts. Red dots are UPR and ER stress‐related genes. The UPR and ER stress genes significantly down‐regulated only under low glucose conditions in sh*Cct3* cells compared to controls are indicated near the lower graph. For better visualization, two dots with *q* ≤ 0.001 have been removed from the volcano plot (see them in Dataset EV4). Y‐axis indicates the false discovery rate (FDR) (*q*‐value), and the X‐axis indicates the log2 fold changes. The FDR‐based method of *P*‐value adjustment was conducted to calculate the q‐values by nSolver software using Benjamini–Hochberg methods. Significance is established by *q*‐value < 0.05 and indicated by the dotted horizontal line. *n* = 3 mice.B“UPR and ER stress” genes are significantly enriched (only down‐regulated) in sh*Cct3* cells compared to controls under low glucose conditions (**P < 0.01 using two‐sided Chi‐square test).CThe graph shows that the ratio between *Xpb1*(spliced) / *Xpb1*(unspliced) is lower (i.e. lower levels of UPR) in LSK which have *Cct‐3* down‐regulated compared to controls only when cultured under low glucose conditions. Error bars are SD from the mean. *P* < 0.001 (three asterisks), and n.s. indicates not significant using two‐tailed Student's test with equal sample variance. *n* = 3 mice. Source data are available online for this figure.

To test whether *Cct3*‐mediated UPR may be important in a mammalian regenerative system, HSPCs (LSK cells: Lineage^−^, Sca‐1^+^, c‐Kit^+^ cells) (Okada *et al*, 1992) from mouse bone marrow were lentiviral transduced with a *Cct3‐*shRNA or a scramble control shRNA. Transduced LSK cells were either cultured under normal (6 mM) or low (2 mM) glucose conditions. NanoString analysis of stress response genes showed that *shCct3* transduction in glucose‐deprived LSK cells resulted in the regulation of genes involved in "UPR and ER genes" (Fig 6A). 32% of the “UPR and ER genes” in the dataset were significantly down‐regulated, which included *BiP* and *Xbp1* (Fig 6B, Dataset EV4). In agreement with the expression data, *Cct3*‐knockdown also decreased the ratio of *Xbp1*(spliced)/*Xbp1*(unspliced), a well‐known marker of ER stress signalling (Yoshida *et al*, 2001), in glucose‐deprived LSK cells (Fig 6C). In line with the planarian data, *Cct3*‐knockdown did not affect the expression of UPR‐related genes or the ratio of *Xbp1*(spliced)/*Xbp1*(unspliced) in LSK cultures exposed to normal glucose conditions.

Together these data support the conclusion that the induction of the UPR and ER stress responses in murine LSK cells in low glucose conditions are dependent on *Cct3*, the murine homologue of *Smed‐cct3A*.

### *cct3A(RNAi)* planarians have decreased energy levels and their regenerative defects can be rescued by supplementation with fatty acids

**Figure 7:**
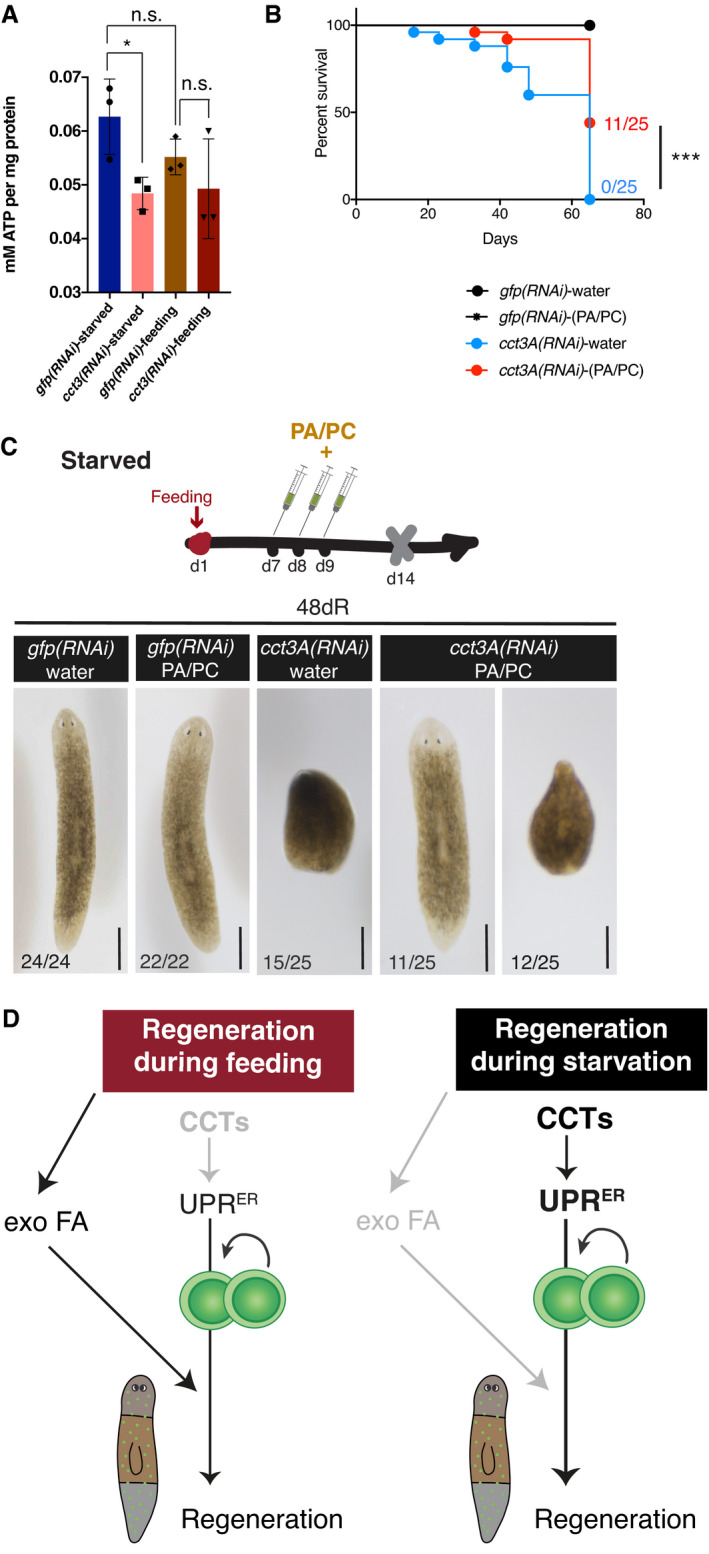
*cct3A(RNAi)* shows decreased ATP levels during starvation and fatty acid administration rescues regeneration defects in a percentage of planarians AATP measurement on 48hR trunk extracts shows that *cct3A* RNAi under starved conditions contains less ATP than controls. Error bars are SD from the mean and asterisks indicate *P* < 0.05 (one asterisk) and n.s. indicates not significant using two‐tailed Student’s test with equal variance. *n* = 3 replicates (5 planarians per replicate).BThe Kaplan–Meier curve demonstrates increased survival of starved *cct3A(RNAi)* animals when treated with palmitic acid/palmitoylcarnitine (PA/PC). Percentages indicate the number of survivals at the indicated time points. Three asterisks indicate *P* < 0.001 with log‐rank (Mantel‐Cox) test.CImages are representative surviving animals for the different conditions in (B). At the bottom are the number of planarians with the phenotype shown. The rest of planarians are dead at this point. Scale bars indicate 500 µm.DIn our proposed model the levels of ER stress (UPR) are basal during regeneration in feeding conditions at 72hR and not essential for stem cell proliferation and regeneration. Planarians obtain energy and lipids from administered food, including exogenous fatty acids (exo FA). During starved conditions, *ccts* increase the levels of the UPR and this allows proper stem cell proliferation and regeneration when exogenous fatty acids are not available. Source data are available online for this figure.

In order to explore the dependency of *ccts* RNAi phenotypes on starvation, we determined general ATP levels in whole planarians (Fig 7A). We observed that controls in starved conditions had ATP levels comparable to controls that were fed. Remarkably, *cct3(RNAi)* animals showed decreased levels of ATP specifically in starved conditions when compared to controls (Fig 7A), indicating that *cct3A* is required to maintain ATP levels in starved planarians.

We reasoned that if *cct3A(RNAi)* planarians were deficient in energy, supplementation with a dietary source of fuel such as fatty acids could rescue *cct3A(RNAi)* phenotypes. We injected palmitic acid (PA), one of the most abundant fatty acids in animals, and its mobilized form palmitoyl‐*L*‐carnitine (PC) before RNAi injection in starved conditions as done previously (Deb *et al*, 2021). Interestingly, PA/PC significantly increased the survival of *cct3A(RNAi)* planarians (Fig 7B and C). While all non‐supplemented *cct3A(RNAi)* planarians did not regenerate or were dead by 48dR, 44% of the PA/PC supplemented *cct3A(RNAi)* planarians were able to fully regenerate (Fig 7B and C) and survived at least until 65dR when all the non‐supplemented *cct3A(RNAi)* planarians had died.

Together, the data show that fatty acid supplementation is able to revert the regenerative failure of *cct3A(RNAi)* planarians under starved conditions implying that lipid metabolism is a downstream target of *cct3A* required for the maintenance of regeneration in starved conditions.

## Discussion

In this study, we found CCT‐mediated UPR modulation as a mechanism that contributes to explain how planarians can regenerate under starved conditions. CCT subunits function as a multiprotein complex called the TRiC, acting as a chaperonin in the process of correct protein folding. It is known that all eight subunits are required for the proper function of TRiC, and depletion of any subunit is able to reduce its activity (Tam *et al*, 2006). However, free monomeric subunits have been described as having some additional roles outside the CCT oligomer (Vallin & Grantham, 2019). Indeed, using siRNAs to reduce levels of CCT subunits disrupts both CCT oligomer functions and functions associated with specific CCT monomeric forms (Vallin & Grantham, 2019). Also, depleting one CCT subunit results in a reduction of assembled oligomer and that in turn increases monomeric non‐targeted CCT subunits. It has been predicted that if targeting different CCT subunits gives the same results, then most likely the oligomer function has been affected (Vallin & Grantham, 2019). Our experiments show the same phenotype after independently down‐regulating each of the 8 subunits, and we also see an increase of the non‐targeted *ccts* mRNAs after *cct3A* down‐regulation (Fig 4A), which could be explained by a feedback loop of the cell trying to re‐establish sufficient TRiC levels. Therefore, it is likely that the role of CCTs on planarian regeneration during starvation depends on the whole complex rather than on CCT subunits acting as monomers.

How TRiC subunits or the TRiC in the cytosol is able to regulate the stress response at the ER and whether the TRiC itself is responding to stress will require further research. The protein HSF1, a transcription factor that responds to stress to protect cells from protein misfolding and that is known to be directly regulated by the TRiC (Neef *et al*, 2014), could represent a possible link. Alternatively, the TRiC itself, or a TRiC client or TRiC subunits may be able to bind to ATF6 which would explain *xbp1* and *bip* modulation.

Our experiments suggest that the effects we observe after down‐regulation of *ccts* are mainly due to CCTs regulating the UPR in stem cells. In line with previous reports on mammalian stem cells (Noormohammadi *et al*, 2016), we found that planarian stem cells show increased expression of several chaperones including all TRiC subunits when compared to differentiated cells. We also showed that *bip,* a well‐known ER chaperone which is transcriptionally regulated in response to ER stress, is strongly down‐regulated in S and G2/M stem cells (X1 fraction) after *cct3A* or *cct4B* RNAi. Furthermore, *cct3A* RNAi and *xbp1/atf6* RNAi shows stem cell‐related phenotypes (defects in mitosis, increased apoptosis during regeneration which includes X1 stem cells and stem cell depletion in case of *cct3A* RNAi) and prevent regeneration. Mild DTT treatment on whole planarians greatly affects stem cells, since it enhances planarian regeneration of *cct3A(RNAi)* planarians in starving conditions and indeed allows for survival of the lethal *cct3A* RNAi. As a way to complement our results with an additional regenerative system which does not have limitations regarding UPR assays, we found that mouse *shCct3* hematopoietic stem cells and progenitor cells, when grown under low glucose conditions, functionally down‐regulate the UPR. Altogether the data suggest that the observed phenotypes are predominantly due to CCT regulating the UPR in X1 stem cells. However, because *ccts* and more importantly *xbp1* and *atf6* are also expressed in the X2 (G0 and G1 stem cells) and Xins (differentiated cells), we cannot formally rule out the possibility that part of the phenotype is due to defects in differentiating postmitotic and/or differentiated cells.

The human chaperome is formed by 332 chaperones and co‐chaperones, and each family of chaperones is essential for cell viability indicating that they have non‐overlapping functions (Brehme *et al*, 2014). Indeed, some other planarian chaperones have been linked to regeneration and stem cells (*prohibitin‐1* and *prohibitin‐2*, *mortalin*, *hsp40* and *hsp60*) (Conte *et al*, 2009; Fernandez‐Taboada *et al*, 2011; Rossi *et al*, 2014; Wang *et al*, 2019) and our data validate their enrichment in stem cells. It would be interesting to know whether there are other chaperones apart from CCT subunits which are specifically required for regeneration during starvation. Our cluster 1 data set predicts that at least *prohibitins* (Rossi *et al*, 2014) and one *hsp40* (Fernandez‐Taboada *et al*, 2011) (Dataset EV2a) could also have a nutrient‐dependent effect on regeneration. Our work establishes planarians as an excellent *in vivo* model to investigate functions of proteins in metabolism.

Mechanistically, we found that *cct3A*, a TRiC subunit, is necessary to prevent mitotic defects during the response of stem cells to amputation. *cct3A* RNAi leads to gross nuclear alterations, resulting in cell death observed at wound sites at 4dR. These results highlight the importance of proteostasis to maintain genome integrity. Although it is known that altered proteostasis and genome instability are linked to an increased risk of cancer (Adams *et al*, 2015; Dai & Sampson, 2016), it is not known whether proteostasis is essential for protecting the genome. There are only few evidences indicating a possible role of proteostasis in regulating genome stability. For instance, it has been reported that ER stress and thus activation of the UPR^ER^ induced by tunicamycin or glucose deprivation, suppresses DNA double‐strand break repair in cancer cells by stimulating the degradation of Rad51 (Yamamori *et al*, 2013). Also, Hsp70 has been linked to genome stability in mouse embryonic fibroblasts under heat shock stress (Hunt *et al*, 2004) and Hsp110 is associated with genome instability in cancer cells (Dorard *et al*, 2011). Interestingly, recent studies in planarians and *Drosophila* have shown that the heat shock protein DNAJA1 and HSP90 interact with PIWI proteins suggesting a possible role of these chaperones in suppressing transposition (Gangaraju *et al*, 2011; Wang *et al*, 2019). Although the TRiC has a potential role in cancer development by modulating the folding of client proteins related to oncogenesis, cell cycle and cytoskeleton (i.e. actin and tubulin) (Roh *et al*, 2015), a direct role in genome stability of stem cells is still missing. Since *cct3A* RNAi alters mitosis specifically in starving conditions, it remains possible that TRiC‐dependent protein folding is required for the timed expression of mitosis regulating proteins, especially under starvation. In support of this possibility, we found that the *cyclin‐dependent kinase 1* (*cdk1*) is down‐regulated upon *cct3A(RNAi)* (Dataset EV3c). While our results cannot proof this concept, we think that is an interesting possibility that could contribute to the observed phenotypes. Remarkably, double RNAi for two of the main transcription factors of the UPR^ER^ *xbp1* and *atf6* phenocopies some of the features of *cct3A* RNAi in terms of genome instability. This altogether indicates that one of the major outcomes after *ccts* or the UPR^ER^ down‐regulation during starvation is related to genome integrity in stem cells. Moreover, we show that starvation protects the planarian stem cell genome and thus possibly contributes to the capacity of planarians to circumvent cancer. These results also suggest that the protective effect that fasting confers to stem cells upon chemotherapy (Nencioni *et al*, 2018) could be related to increased proteostasis.

It is known that lipids in the form of lipid droplets (LDs) accumulate under various stress conditions. For instance, mice injected with the ER stress inducer tunicamycin develop hepatic steatosis which is even more severe and showing LD accumulation when any of the UPR branches is knocked down (Rutkowski *et al*, 2008; Yamamoto *et al*, 2010). Several of our results suggest a role of TRiC subunits in lipid metabolism. One single feeding during the fasting period is sufficient to prevent any of the *ccts RNAi* phenotypes and allows regeneration thus indicating that energy production and/or the supply of nutritional building blocks for cellular anabolism prevents the phenotype. Indeed, the administration of fatty acids (PA and PC) increases survival and prevents *cct3A(RNAi)* phenotype of regeneration impairment in a percentage of planarians. Also, *cct3A* RNAi lowers ATP levels which overall suggest that starved *cct3A(RNAi)* planarians cannot generate sufficient energy. One possibility is that *cct3A(RNAi)* planarians in starved conditions fail to provide sufficient fatty acids and/or cholesterol for membrane expansion in order to pass mitosis, a highly energy‐demanding process (Kalucka *et al*, 2015). Future research is needed to clarify the link of TRiC components with lipid and LD metabolism and to elucidate whether this occurs downstream of the UPR.

We propose a model where TRiC components are up‐regulated in response to starvation and this stress response in turn activates the UPR^ER^ that enhances the regenerative capacity. The activation of this protective pathway allows mitotic fidelity and induces a metabolic adaptation of starved animals possibly by providing energy and/or lipid membrane components required for cell growth and proliferation (Fig 7D). During feeding the levels of ER stress are basal and not necessary for the mitotic regenerative response. Energy and lipid components are obtained from exogenous food (Fig 7D). However, we also observed that ER stress induction by DTT treatment in fed *cct3A* RNAi planarians but not in controls led to a decrease in survival. These data suggest that *cct3A* RNAi also has effects under feeding conditions that *per se* do not disturb regeneration but synergizes with DTT‐mediated induction of ER stress/UPR to impair survival. While this mechanism remains to be elucidated, the data on starved planarians provide experimental evidence that *cct3A*‐dependent induction of ER stress response/UPR is required for regeneration under starvation conditions.

## Materials and Methods

### Planarian husbandry

Planarians used in this work belong to the species *S. mediterranea* asexual biotype. All animals were maintained at 19°C in 1× Montjuïc Salts (1.6 mM NaCl, 1 mM CaCl_2_, 1 mM MgSO_4_, 0.1 mM MgCl_2_, 0.1 mM KCl, 0.1 g NaHCO_3_ per litre) and fed with organic veal liver.

### Starvation and feeding experiments

In experiments involving feeding, all animals were observed to make sure they ate. Food was given in excess and removed after 2 h. For RNA‐seq experiments, planarians at 1dS, 7dS and 30dS had the same area 4 mm^2^ (5–5.5 mm length at 7dS and 1dS and 5.5‐ 6mm length at 30dS). Graph paper placed under the Petri dish was used to pre‐select animals, and the final selection was done after measuring the areas with the Leica Application Suite (Leica) on photographs of live planarians taken under a stereomicrospe coupled with a Leica camera MC170 HD (Leica). For RNAi experiments, planarians around 5 mm were selected by use of graph paper.

### Fluorescence‐activated cell sorting (FACS)

Planarian dissociation and cell population analysis were performed as described previously (Hayashi *et al*, 2006). Briefly, planarians were cut into small pieces on ice and cell dissociated in the presence of papain (Merck; final concentration 1 mg/ml) during 15 min at room temperature (Moritz *et al*, 2012). Cells were then filtered through a 40 µm filter, counted and resuspended in staining solution containing the cytoplasmic dye Calcein‐AM (Biotium; final concentration of 0.5 µg/ml) and the nuclear dye Hoechst 33342 (Thermo Fisher Scientific; final concentration 30 µg/ml) in order to isolate X1, X2 and Xins populations. Staining was performed in the dark at 25°C, with continuous shaking. Propidium iodide (final concentration 1 µg/ml) was added 1 min before flow cytometry analysis to discard dead cells. Around 250,000 events were sorted per sample using BD FACSAria III or BD FACS Fusion. Cells were put into tubes containing TRIzol LS (Ambion), and RNA was extracted following manufacturer’s instructions.

### RNA‐seq

Library preparation was done using the Illumina kit TruSeq. Sequencing was done on an Illumina HiSeq2500 in 51 cycle, single‐end, high‐throughput mode at the Core facility DNA sequencing at FLI. 4 replicates per FACS population (X1, X2 and Xins) and per time point (1dS, 7dS and 30dS) were sequenced: 36 samples (2,304 million of non‐ribosomal reads). Three replicates per RNAi condition (*cct3A(RNAi)* and *gfp(RNAi)* were sequenced: six samples (256 million of non‐ribosomal reads). For transcriptomic analysis, the Dresden *Schmidtea mediterranea* transcriptome (version 4) was used as reference (PlanMine) (Rozanski *et al*, 2019). Kallisto (version 0.43.0) (Bray *et al*, 2016) was used to perform read pseudo‐alignment and quantification with the parameters –l 200 –s 20 –b 100. Previously, only reads mapping to the planarian reference transcriptome were extracted by using Kallisto and the parameter ‐F 4. The portion of planarian ribosomal RNA contamination was identified by mapping all reads with Bowtie2 (Langmead & Salzberg, 2012) against a pool of Platyhelminthes rRNA index. IDs numbers from Dresden transcriptome of version 4 are equivalent to version 6 (Planmine). For example, dresden_comp15_c0_seq1 is equivalent to dd_Smed_v6_15_0_1. Differential expression analysis was performed with the R package Sleuth (version 0.28.1) (Pimentel *et al*, 2017) with the filtered reads mapping to the reference transcriptome. Wald test was used to identify differentially expressed genes (DEGs). Significance was determined by q‐value (false discovery rate (FDR) < 0.1 for pairwise comparisons for the different time points of starvation (7dS versus 1dS, 30dS versus 1dS and 30dS versus 7dS) in the X1. For pairwise comparisons, X1 versus Xins at 1dS, 7dS and 30dS, all TPMs were normalized including also X2 values and the significance was determined by *q*‐value < 0.01. Significance was determined by *q*‐value < 0.05 in the *cct3(RNAi)* RNA‐seq analysis. Downstream analysis was done with the R software. The heat map was done with the R package “Pheatmap”. Clustering was performed using the software package Mfuzz (Kumar & Futschik, 2007). Genes with a value lower than 0.35 for cluster assignment are not displayed in the plots. Gene ontology enrichment was done creating lists of DEGs in PlanMine by using default parameters (test Benjamini–Hochberg; *P* < 0.05). Redundant GO terms were removed from the list by using REVIGO with small similarity parameter (Supek *et al*, 2011).

### RNAi experiments

Templates with T7 promoters appended to both strands were generated. Double‐stranded RNA (dsRNA) was synthesized by *in vitro* transcription following MEGAscript RNAi kit (Ambion) instructions. dsRNA was injected into the planarian as previously described (González‐Estévez *et al*, 2012b). Control animals were injected with *gfp* dsRNA, a sequence not present in the planarian genome. The different protocols used are specified in the “Results” section. The following oligos were used to generate the templates for dsRNA production:

Smed‐cct1A‐F: 5′‐ACCTGGCTATTGGTGGAGAAAG‐3′
Smed‐cct1A‐R: 5′‐GGCATTTGTTGGGAAGCTATTG‐3′
Smed‐cct2‐F: 5′‐TGACAACCCTGCTGCTAAAATC‐3′
Smed‐cct2‐R: 5′‐TGGCAATCTTTTCGACCTTTTC‐3′
Smed‐cct3A‐F: 5′‐CGTCGTTTTGAGTGGAGTTTTG‐3′
Smed‐cct3A‐R: 5′‐TTGATATTGCCATCTCCAATGC‐3′
Smed‐cct4B‐F: 5′‐CTCCAATAGCAGTTGATGCAG‐3′
Smed‐cct4B‐R: 5′‐GGCCAGGATTAACAATTCCACT‐3′
Smed‐cct5‐F: 5′‐TCACTCGGACCAAAGGGATTAG‐3′
Smed‐cct5‐R: 5′‐TGGAGGTTCAAAAGCACAAGTC‐3′
Smed‐cct6‐F: 5′‐GAGTACGCAAAGTCGCAAAATG‐3′
Smed‐cct6‐R: 5′‐TTTCGGCATATCTGGATGTCTG‐3′
Smed‐cct7‐F: 5′‐AAATGTGCTTCCACTGCTCTCA‐3′
Smed‐cct7‐R: 5′‐ACCGCTCTGTTTCCTCCATAAA‐3′
Smed‐cct8A‐F: 5′‐AATGGCTGCTCAACAACAAGAG‐3′
Smed‐cct8A‐R: 5′‐TGCGTTTCTTCTCCACGACTAA‐3′
Smed‐xbp1‐F: 5′‐TAGGTGGGAATGGTATGGGAAA‐3′
Smed‐xbp1‐R: 5′‐CACAACCAAACTCTGACATTTCG‐3′
Smed‐atf6‐F: 5′‐AAGCCAGTTGTTAAGCCAGAAA‐3′
Smed‐atf6‐R: 5′‐CCATGATAACCGGGAAATGAAGA‐3′
Smed‐bip‐1‐F: 5′‐ TGTTCTTGTCGGTGGTTCAACT −3′
Smed‐bip‐1‐R: 5′‐ AGCGCCCAATTTTTCTTTATCA −3′
Smed‐bip‐2‐F: 5′‐ AAATCCGAACCAAAGGAACATC −3′
Smed‐bip‐2‐R: 5′‐ GTTTCGCCATTACTCCACCTTC −3′
Smed‐bip‐3‐F: 5′‐ GGAGGGACATTTGATGTTTCGT −3′
Smed‐bip‐3‐R: 5′‐ TCATTACTGCGCCAACAGTTTC −3′

*Smed‐smg‐1* and *Smed‐tor* oligos were as previously published (González‐Estévez *et al*, 2012b). The sequence of *cct8A* has been previously deposited in GenBank: MF669570 (Counts *et al*, 2017).

### Immunohistochemistry

Whole‐mount immunohistochemistry was performed as previously described (Cebrià & Newmark, 2005). The following primary antibodies were used: anti‐VC‐1 (Sakai *et al*, 2000) (diluted 1/15,000; kindly provided by Professor K. Watanabe and H. Orii); anti‐TMUS13 (Cebrià *et al*, 1997) (diluted 1/20; kindly provided by Professor R. Romero); anti‐acetylated‐tubulin (Iglesias *et al*, 2011) (diluted 1:200, Sigma; clone 6‐11B‐1); and anti‐Histone H3 phosphorylated at serine 10 (H3P, diluted 1/500, Santa Cruz; sc‐8656‐R). Nuclei were stained with DAPI (1 µg/ml). Double immunohistochemistry on dissociated cells was performed as previously described (de Sousa *et al*, 2018). An anti‐Histone H3 phosphorylated at serine 10 (H3P, diluted 1/500, Santa Cruz; sc‐8656‐R) and anti‐Tubulin Tyrosin (diluted 1/400; Sigma; T9028) were used.

### Real‐Time PCR (qPCR)

RNA was extracted using TRIzol (Ambion) for whole planarians and TRIzol LS (Ambion) for FACs sorted cells. cDNA was obtained from 200 ng (from sorted cells) or 1 µg (from whole animals) of total RNA by using MMLV Reverse Transcriptase (Promega). As internal control Elongation Factor 2 (EF2), the transcript with ID 5685 from Dresden transcriptome (Planmine) or act‐like (SMEST057988001.1) were used. Each qPCR was performed with three biological replicates. Five animals were used per replicate, and each sample was replicated three times in each real‐time PCR experiment. PCR reactions were performed using the iTaq Universal SYBER^®^ Green Supermix (BIO‐RAD). Reactions were aliquoted using a QiAgility robot (Qiagen) and analysed with a 7500 Real‐Time PCR System (Applied Biosystems). The following gene‐specific oligos were designed from regions of the gene that were non‐overlapping with the sequence used for dsRNA:

SmEf2‐F: 5′‐CAGCCAGTAGCTTTAAGCGATGA‐3′
SmEf2_R: 5′‐ACTCTCAACGCTGCTGTCACTTC‐3′
5685_qPCR1: 5′‐CTTCAAGTCAAAGCATCATTTACTC‐3′
5685_qPCR2: 5′‐CATTTCGCAAATCATCTTCTAACTG‐3′
qWi1PPF: 5′‐GCAGAGAAACGGAAGTAATAGAG‐3′
qWi1PPR: 5′‐ATCCAATCCTACAATCATAGTCGG‐3′
xbp1‐QF: 5′‐ATTGGAAACTCCCATTTGTGAC‐3′
xbp1‐QR: 5′‐AGAATTATCTGGCTGACTTTGG −3′
atf6‐QF: 5′‐ TAAGAAATCGAAATTCCGGGAC‐3′
atf6‐QR: 5′‐ AAGCGTATAATCCTTGGTTCTG‐3′
cct3A_QF: 5′‐ GCAATATCAACTCACTTGACCCT‐3′
cct3A_QR: 5′‐ TAACCTTAACACCTTTGCTCCA‐3′
cct4B_QF: 5′‐ CAGAGTTAAGAAATAGACATGCCAG‐3′
cct4B_QF: 5′‐ ACTGTGATTGATAAGGAGTGGT‐3′
cct7_QF: 5′‐ AATTCCACGACAATTATGCGAG‐3′
cct7_QR: 5′‐ CTTCATTAAGAATGTCGACTCCAC‐3′
bip1_QF: 5′‐ATGAAATTGTTCTTGTCGGTGG‐3′
bip1_QR: 5′‐CTGCTTCATCTGGATTAATACCTC‐3′
act_QF: 5′‐GTAGCTCCAGAAGAACATCCAG‐3′
act_QR: 5′‐CAGAAGCATACAAAGACAACACAG‐3′

### Whole‐mount *in situ* hybridization

Whole‐mount *in situ* hybridization was carried out as described previously (González‐Estévez *et al*, 2012b) using an InsituPro VSi (Intavis). Hapten‐labelled RNA probes were prepared by using an *in vitro* RNA labelling kit (Roche).

### Measurement of ATP levels

ATP levels were measured by using an ATP bioluminescence assay kit HS II (Roche) according to the manufacturer’s instructions. Regenerating trunks were incubated in cell lysis reagent and boiled for 15 min at 100°C. Then, samples were sonicated for 4 cycles of 30′′. Luciferase reagent was added to the samples, and luminescence was measured by the Mithras LB940 plate reader (Berthold Technologies) using the injection function. Three biological replicates consisting of five trunks at 48hR were used per condition, and each sample was replicated three times in each ATP experiment. Protein content was measured by the Bradford method (Bio‐Rad Protein Assay).

### Whole‐mount TUNEL

Whole‐mount TUNEL was performed as previously described (Stubenhaus & Pellettieri, 2018).

### Imaging and quantifications

Z‐stacks were acquired with a Zeiss ApoTome.2 equipped with a Zeiss Axiocam 503 mono (Carl Zeiss, Jena) (immunohistochemistry on dissociated cells), a Zeiss LSM 710 ConfoCor 3 microscope (Carl Zeiss, Jena) (whole‐mount immunohistochemistry and whole‐mount TUNEL) or a Zeiss AXIO Zoom.V16 (Apotome.2) equipped with Axiocam 506 mono and colour (Carl Zeiss, Jena) (H3P whole‐mount immunohistochemistry and whole‐mount FISH). Images were processed using Fiji (Schindelin *et al*, 2012) and Adobe Photoshop CS6/Adobe CC software. H3P quantifications were done on whole planarians using Object Counter 3D plugin from Fiji (Schindelin *et al*, 2012).

### Annexin V staining

Annexin V staining was performed according to previously published (Shiroor *et al*, 2020) with the following modifications. After staining with Hoechst 33342 and Calcein‐AM (as previously explained for the FACS protocol), 500,000 and 2 × 10^6^ cells/sample were pelleted and stained with APC Annexin V RUO (BD Pharmingen; 550475) in a ratio of 5 µl Annexin V: 100 µl freshly made 1X Annexin V buffer (10× concentrate RUO, BD Pharmingen): 100,000 cells. After staining for 15 min in the dark at 25°C, cells were washed and suspended in 300 µl of 1× Annexin V buffer with 1 µg/ml Propidium Iodide and analysed on BD FACSAria III. FlowJo™ Software (FlowJo 10.6.2, Ashland, OR) was used to analyse the data.

### DTT treatment

DTT at concentrations 0.05, 0.1 and 0.2 mM was added to the planarian water, and planarians were incubated with that solution for the 3.5 h previous to each RNAi injection. In experiments on non‐RNAi planarians, the incubations were performed for 3.5 h on the days that RNAi injections would occur.

### Administration of palmitic acid (PA) and palmitoylcarnitine (PC)

12mM BSA‐conjugated palmitic acid was prepared as previously (Deb *et al*, 2021). Briefly, 12 mM palmitic acid was prepared by mixing palmitic acid (Sigma‐P0500) in EtOH 100%. Vortex was required to completely dissolve it. Then, 0.2680 gr of fatty acid‐free BSA was resuspended in 2 ml of PBS (pH 7.2) at 37°C and subsequently treated in an ultrasound bath for 10 min at 37°C. Next, palmitic acid solution was pre‐warmed at 37°C for 2 min and 37 µl was added very slowly (in drops of 7 µl) to the fatty acid‐free BSA mixture. Vortex was required after every drop. The 12 mM BSA‐conjugated palmitic acid solution was filtered through a 0.2‐µm mesh. 11 mM palmitoyl‐*L*‐carnitine chloride (Sigma‐P1645) was prepared in water. A solution containing 750 µM BSA‐conjugated palmitic acid and 750 µM palmitoyl‐*L*‐carnitine chloride was freshly prepared every day of injection. Three pulses of 69 nl were injected into planarians 3 h prior to RNAi injections.

### Experiments with mice

Mice used in this study were C57BL/6J wild‐type mice (5‐ to 6‐month‐old) obtained from Janvier or from internal stock (FLI internal license O_KLR_18‐20 and §11 Lizenz). Mice were maintained in a specific pathogen‐free animal facility in Fritz Lipmann Institute with 12 h of light/dark cycle and fed with a standard mouse chow. Animal experiments were performed according to Institutional and European Union guidelines.

### Isolation of mouse bone marrow cells and FACS sorting of hematopoietic stem and progenitor cells (LSK cells: Lineage^−^, Sca‐1^+^, c‐Kit^+^ cells)

Total bone marrow was isolated from bones (tibia, femur, pelvis and vertebral column). Cells were then stained with anti–c‐Kit‐APC (BioLegend; 105812) antibody at 4°C for 30 min. c‐Kit^+^ cells from total bone marrow (BM) were enriched through magnetic‐activated cell separation (MACS; Miltenyi Biotec) according to the manufacturer’s protocol (LS Column, 130‐042‐402; anti–APC‐microbeads, 130‐090‐855). Cells were incubated with a lineage cocktail containing biotinylated antibodies against CD4 (BioLegend; 100508), CD8 (BioLegend; 100704), TER‐119 (BioLegend; 116204), CD11b (BioLegend; 101204), Gr‐1 (BioLegend; 108404), and B220 (BioLegend; 103222) at 4°C for 30 min. After washing, cells were incubated with anti–Sca‐1‐PE‐Cy7 (BioLegend; 108114) and anti–c‐Kit‐APC (BioLegend; 105812) antibodies. Biotinylated antibodies were developed with streptavidin‐APC‐Cy7 (BioLegend; 405208). LSK cells, which contain hematopoietic stem cells (HSCs) and multipotent progenitors (MPPs), were FACS sorted by selecting for Sca‐1^+^, c‐Kit^+^, lineage^−^ cells.

### Lentivirus production

shRNA was inserted into the SF‐LV‐shRNA‐EGFP plasmid using mir30 primers (Chen *et al*, 2019). Lenti‐X (Clontech) cells were cultured in DMEM supplemented with 10% FBS, 100 U/ml penicillin and 100 µg/ml streptomycin. Lentivirus was produced in Lenti‐X cells using calcium phosphate transfection of 30 µg shRNA plasmid, 18 µg psPAX2 and 9 µg pMD2.G plasmids according to standard procedures (Schambach *et al*, 2006). Medium was changed 12 h after transfection and virus supernatant was collected 36 h after changing medium. Lentiviruses were concentrated at 106,800 *g* for 2.5 h at 4°C, and viral pellets were resuspended in sterile PBS.

*Luciferase* shRNA: 5′TGCTGTTGACAGTGAGC GCCCGCCTGAAGTCTCTGATTAATAGTGAAGCCACAGATGTATTAATCAGAGACTTCAGGCGGTTGCCTACTGCCTCGGA‐3′
*Cct3* shRNA: 5′TGCTGTTGACAGTGAGCGCACGTGGAGTTATGATTAACAATAGTGAAGCCACAGATGTATTGTTAATCATAACTCCACGTATGCCTACTGCCTCGGA‐3′

### NanoString analysis on normal and low glucose cultured LSK cells

Measuring of RNA expression by NanoString was done according to previously (Chen *et al*, 2019) and to manufacturer protocol (NanoString Technologies). In brief, 5 × 10^4^ hematopoietic stem and progenitor cells (LSK cells) were transduced with shRNA targeting murine *Cct3* or *luciferase* control. The cells from both conditions were then cultured in DMEM medium (Gibco; A1443001) with low glucose concentration (2 mM) or normal glucose concentration (6 mM) for 16hrs. Both of the culture conditions contained 4 mM glutamine, 50 ng/ml mTPO (Peprotech; 315‐14), 50 ng/ml SCF (Peprotech; 250‐03), 100 unit/ml penicillin and 100 µg/ml Streptomycin (Gibco; 15140‐122). 1 × 10^4^ cells were lysed in 2 µl of lysis/binding solution (Applied Biosystems; 8500G14). The cell lysate was then used for hybridization reaction as following: 2 µl of cell lysate was mixed with 5 µl of nCounter hybridization buffer (NanoString), 2 µl of Core Tagset, 2 µl of extension Tagset, 0.5 µl of 0.6 nm Probe A working pool, 0.5 µl of 0.6 nm probe A extension Pool, 0.5 µl of 3 nm Probe B working pool and 0.5 µl of 3 nm Probe B extension pool (IDT technologies). 2 µl of Nuclease‐free water was added to each reaction to reach a final volume of 15 µl. The reaction mixture was prepared in Strip tubes (NanoString technologies). Then, it was incubated at 67°C using a thermal cycler for 16 h. The Nanostring chemistry was processed automatically using nCounter prep‐station 5s (NanoString Technologies) according to manufacturer protocol. Directly after the run, the nCounter Cartridge was loaded into nCounter digital analyser 5s (NanoString technologies). Data analysis and *q*‐values were obtained after background correction using nSolver advanced analysis software (v.4) and (R software v 3.3.2.). The following housekeeping genes were used for normalization: ActB, B2 M, Gapdh, Gusb, Hprt, PGK1, Polr1b, Polr2a, Ppia, Rpl19, Sdha and Tbp. For analysis of significantly expressed genes by NanoString, the FDR‐based method of *P*‐value adjustment was conducted to calculate the q‐values by nSolver software (v.4. Based on R‐language) using the Benjamini–Hochberg method. Significance was established by *q*‐value < 0.05. Volcano plots were obtained with GraphPad Prism software version 8. The *Xpb1*(spliced)/*Xbp1*(unspliced) ratio was calculated by dividing the number of mRNA counts of spliced *Xpb1*/unspliced *Xbp1* after normalization to housekeeping genes.

### Statistical analysis in planarian experiments

In all the manuscript, error bars are SD from the mean and asterisks indicate *P* < 0.05 (one asterisk), *P* < 0.01 (two asterisks) or *P* < 0.001 (three asterisks), *P* < 0.0001 (four asterisks) and n.s. indicates not significant using two‐tailed Student’s test with equal variance or two‐sided chi‐square test or log‐rank (Mantel‐Cox) test for Kaplan–Meier curves. The number of replicates is indicated in figures and figure legends. Wald test was used to identify differentially expressed genes (DEGs). Significance was determined by *q*‐value (false discovery rate (FDR)) < 0.1 for pairwise comparisons for the different time points of starvation (7dS versus 1dS, 30dS versus 1dS and 30dS versus 7dS) in the X1. Significance was determined by *q*‐value < 0.01 for pairwise comparisons X1 versus Xins at 1dS, 7dS and 30dS. Significance was determined by *q*‐value < 0.05 in the *cct3(RNAi)* RNA‐seq analysis. More details on the RNA‐seq statistics can be found in the RNA‐seq sections. For analysis of gene expression by NanoString, significance was determined by *q*‐value < 0.05.

## Author contributions

OG‐G, DAF and CG‐E performed most of the experiments with the help of AT; OG‐G performed computational analysis; AK and AS helped in the project; SP performed the clustering analysis; EMA and KLR performed the experiments on mouse hematopoietic stem and progenitor cells; OG‐G, DAF and CG‐E designed experiments and analysed the data; CG‐E directed the project; CG‐E wrote the manuscript with help of OG‐G, DAF, AS, AK and KLR.

## Conflict of interest

The authors declare that they have no conflict of interest.

## Supporting information



Appendix

Expanded View Figures PDF

Dataset EV1

Dataset EV2

Dataset EV3

Dataset EV4

Source Data for Expanded View and Appendix

Review Process File

Source Data for Figure 1

Source Data for Figure 2

Source Data for Figure 3

Source Data for Figure 4

Source Data for Figure 5

Source Data for Figure 6

Source Data for Figure 7

## Data Availability

Sequences have been deposited in GenBank with the accession numbers:
*xbp1*: MN171093 (https://www.ncbi.nlm.nih.gov/nuccore/MN171093)
*atf6*: MN171094 (https://www.ncbi.nlm.nih.gov/nuccore/MN171094)
*cct3A*: MN171095 (https://www.ncbi.nlm.nih.gov/nuccore/MN171095)
*cct1A*: MN171096 (https://www.ncbi.nlm.nih.gov/nuccore/MN171096)
*cct2*: MN171097 (https://www.ncbi.nlm.nih.gov/nuccore/MN171097)
*cct4B*: MN171098 (https://www.ncbi.nlm.nih.gov/nuccore/MN171098)
*cct5*: MN171099 (https://www.ncbi.nlm.nih.gov/nuccore/MN171099)
*cct7*: MN171100 (https://www.ncbi.nlm.nih.gov/nuccore/MN171100)
*cct6*: MN380639 (https://www.ncbi.nlm.nih.gov/nuccore/MN380639) bip‐1: MT411562 (https://www.ncbi.nlm.nih.gov/nuccore/MT411562) bip‐2: MT411563 (https://www.ncbi.nlm.nih.gov/nuccore/MT411563) bip‐3: MT411564 (https://www.ncbi.nlm.nih.gov/nuccore/MT411564) RNA‐Seq data has been deposited in Gene Expression Omnibus (GEO) with the accession numbers:
GSE134148 (https://www.ncbi.nlm.nih.gov/geo/query/acc.cgi?acc=GSE134148)
GSE134013 (https://www.ncbi.nlm.nih.gov/geo/query/acc.cgi?acc=GSE134013)
