# Supplementary material for: Regeneration in starved planarians depends on TRiC/CCT subunits modulating the unfolded protein response
Source: EMBO Rep. 2021 Jun 30;22(8):e52905. doi: 10.15252/embr.202152905 (PMC8344900; doi:10.15252/embr.202152905)
Supplement: Supplementary file 1 — Appendix [file EMBR-22-e52905-s008.pdf]

Table of contents:

Appendix Figure S1..2

Appendix Figure S2..3

Appendix Figure S3..4

Appendix Figure S4..5

A

Trunks 27dR	*gfp(RNAi)* starved	*gfp(RNAi)* feeding	*smg-1(RNAi)* starved	*smg-1(RNAi)* feeding
Normal	100% (18/18)	100% (18/18)	5.55% (1/18)	5.55% (1/18)
Abnormal unpigmented blastemas with or without hyperplasia			66.66% (12/18)	11.11% (2/18)
Abnormal unpigmented blastemas, hyperplasia and/or outgrowths			16.66% (3/18)	22.22% (4/18)
Dead after outgrowths			11.11% (2/18)	61.11% (11/18)

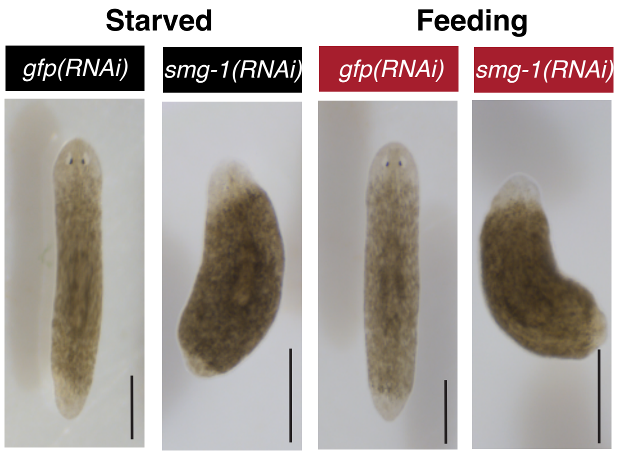

B

Trunks 23dR	*gfp(RNAi)* starved	*gfp(RNAi)* feeding	*tor(RNAi)* starved	*tor(RNAi)* feeding
Normal	100% (11/11)	100% (11/11)		
BLST(0-1.5)			100% (11/11)	
Anterior BLST(0-1.5); Posterior BLST(> 1.5)				27.27% (3/11)
Dead				72.72% (8/11)

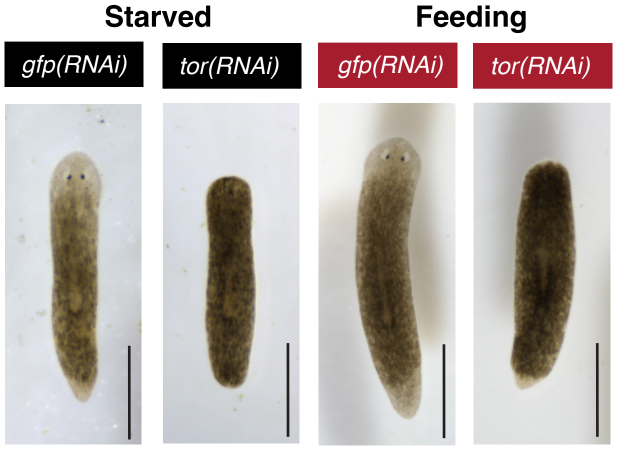

C

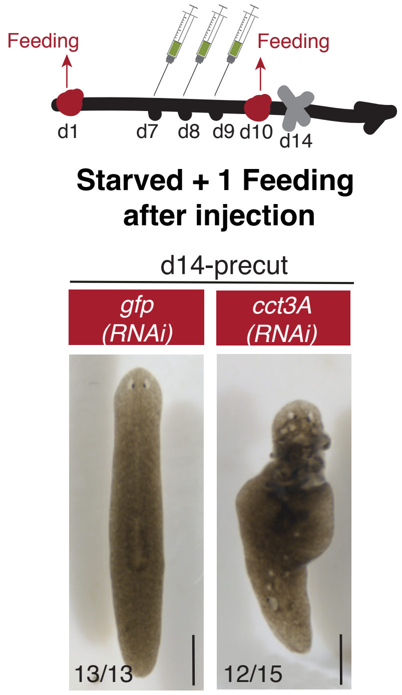

D

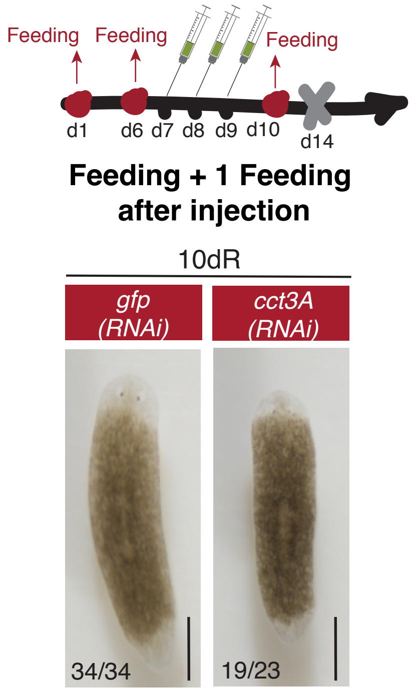

Appendix Figure S1. Controls on the RNAi injections.

(A) Number of planarians displaying the different *smg-1* RNAi phenotypes for the starved and feeding conditions performed with the same RNAi schedules as for *cct* genes. Highlighted in red: the number of dead planarians after outgrowths at 27dR is higher in *smg-1(RNAi)*-feeding than in starved conditions. Representative images displaying the typical phenotype of abnormal unpigmented blastemas in *smg-1(RNAi)* planarians at 20-27dR. The same phenotype is observed with both the starvation and the feeding RNAi schedules. (B) Number of planarians displaying the *tor-1* RNAi phenotype at 23dR for the starved and feeding conditions performed with a similar RNAi schedule as for *cct* genes (feeding is prior to injections, however, the gene requires two rounds of injections and regeneration as previously described (Gonzalez-Estevez et al., 2012b)). Highlighted in red: most of the planarians are already dead by 23dR when *tor(RNAi)* is performed in feeding conditions. BLST indicates blastema size as previously described (Gonzalez-Estevez et al., 2012b). Posterior BLST (>1.5) in 40% of the planarians that died at 23dR. Representative images displaying the phenotype at 23dR. (C) RNAi injections schedule in starving conditions, placing a feeding after injections. Most of *cct3A(RNAi)* display lesions and lysis before amputation at day 14. All these planarians die between day 15 and 18. At the bottom are the number of planarians with the phenotype shown. The remaining planarians were amputated. All the *cct3A(RNAi)* amputated planarians died at 1-2dR. (D) RNAi injections schedule in feeding conditions, placing an extra feeding after injections. Most of *cct3A(RNAi)* planarians have regenerated at 10dR. Note that the percentage of regenerated planarians at 10dR is higher (82.61%) than when the feeding is only placed at day 6 (Fig. 1C; 78.22%). At the bottom are the number of planarians with the phenotype shown. The remaining planarians were dead at the indicated time point. dR, days of regeneration. Scales, 500 μm.

A

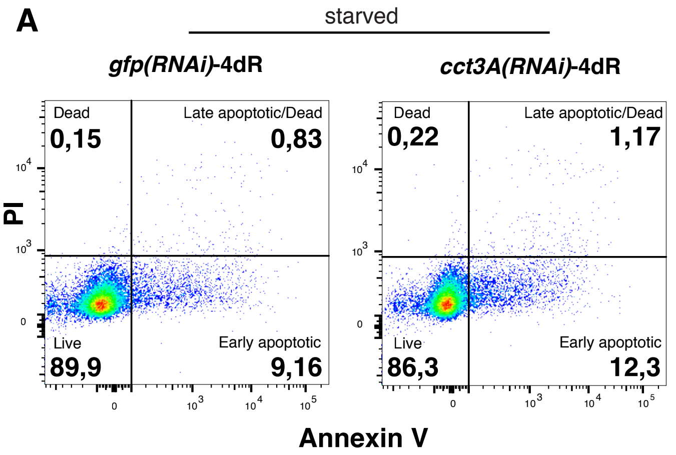

B

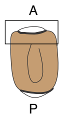

Starved conditions

72hR	Percentage of cells in different mitotic phases		Percentage of defective figures	
	***gfp*(RNAi) n=42**	***cct3A*(RNAi) n=42**	***gfp*(RNAi)**	***cct3A*(RNAi)**
Prophase/ Prometaphase	19.05% (8/42)	21.43% (9/42)	0% (0/8)	88.88% (8/9)
Metaphase	42.86% (18/42)	16.67% (7/42)	0% (0/18)	85.71% (6/7)
Anaphase	38.10% (16/42)	61.90% (26/42)	0% (0/16)	76.92% (20/26)

C

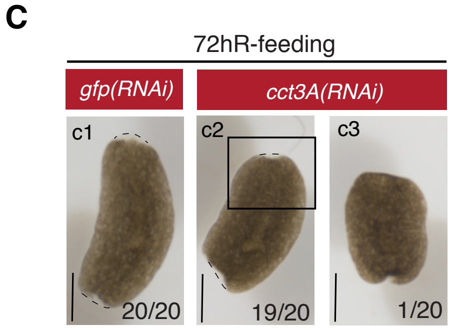

D

Feeding conditions

72hR	Percentage of cells in different mitotic phases		Percentage of defective figures	
	***gfp*(RNAi) n=55**	***cct3A*(RNAi) n=65**	***gfp*(RNAi)**	***cct3A*(RNAi)**
Prophase/ Prometaphase	29.09% (16/55)	36.92% (24/65)	0% (0/16)	33.33% (8/24)
Metaphase	40% (22/55)	40% (26/65)	0% (0/22)	7.69% (2/26)
Anaphase	30.91% (17/55)	23.08% (15/65)	0% (0/17)	33.33% (5/15)

E

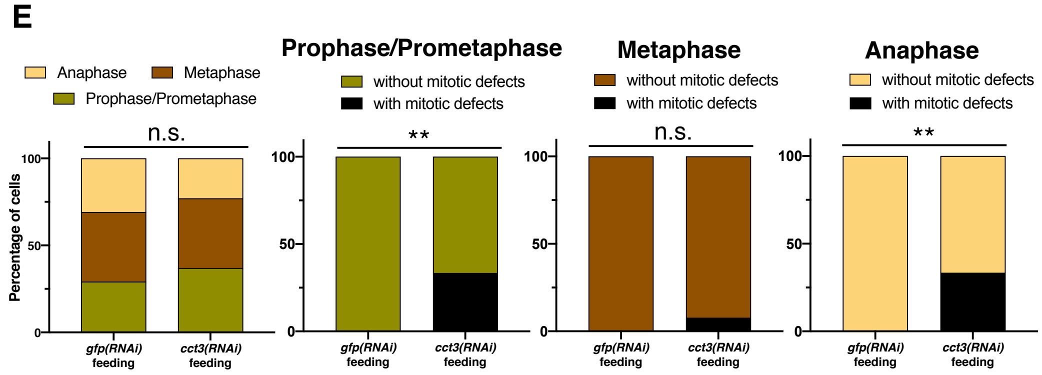

Appendix Figure S2. During starvation stem cells from 72hR *cct3A(RNAi)* planarians show defective mitosis and die, which is partially prevented by feeding.
(A) Representative plot showing living and dead X1 cells (stem cells) stained with propidium iodide (PI) and annexin V after *cct3A(RNAi)* and controls at 4dR. Notice the increase in stem cell death after *cct3A(RNAi)* (sum of annexin V positive compartments: 13.47%) compared to controls (9.99%). Approximately 20,000 X1 cells are displayed. $2x10^6$ total planarian cells were analyzed from 25 planarians per sample in two independent experiments. (B) Quantification of the percentage of stem cells in different mitotic phases and the percentage of defective mitotic figures in 72hR anterior blastemas of *cct3A* RNAi and controls during starvation after double immunostaining with anti-α-tubulin and anti-H3P. The cartoon represents a regenerating trunk and the square displays the region analyzed at the anterior "A" blastema. (C) Representative live images of 72hR trunks from controls and *cct3A(RNAi*) planarians under feeding conditions. From the pool of fed *cct3A(RNAi)* planarians only the planarians with bigger blastemas were selected (c2). From that pool, not all planarians would have finally regenerated as the percentage 95% (19/20) is higher than the one at final stages of regeneration (83.87% in Fig. 1C). The box indicates the region analysed for mitotic figures in D. The dotted line delimits the blastema. At the bottom are the number of planarians with the phenotype shown. Scales, 800 μm. (D) Quantification of the percentage of stem cells in different mitotic phases and the percentage of defective mitotic figures in 72hR anterior blastemas of *cct3A* RNAi and controls during feeding after double immunostaining with anti-α-tubulin and anti-H3P. (E) Quantification of the percentage of stem cells in different mitotic phases and the percentage of defective mitotic figures in 72hR anterior blastemas of *cct3A* RNAi and controls during the feeding conditions after double immunostaining with anti-tyrosine-tubulin and anti-H3P (**P < 0.01 and n.s. not significant using two-sided Chi-square test; number of analyzed cells is displayed in D); n ≥ 5.

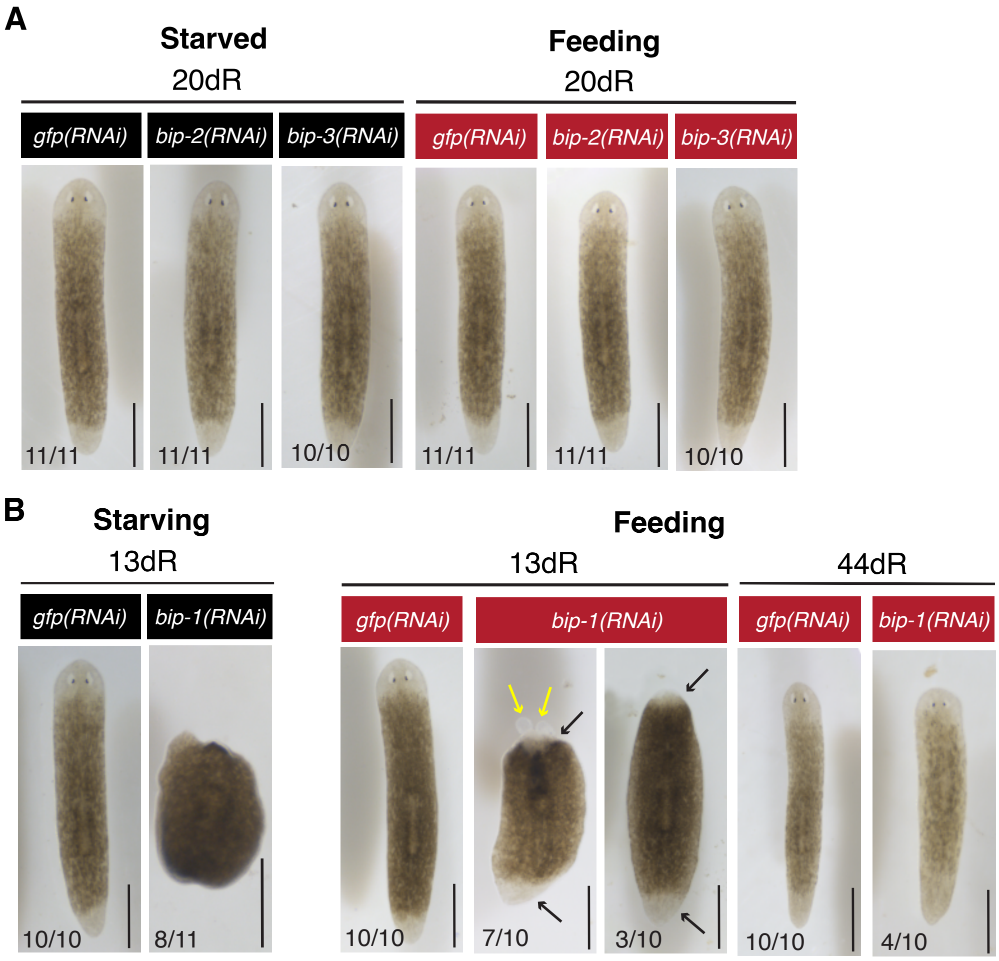

Appendix Figure S3. ***bip-1*** **RNAi phenocopies** ***cct3A*** **RNAi.**
(A) RNAi injections schedule in starved and feeding conditions for *bip-2* and *bip-3* as performed for *cct3A* in Fig. 1. Live images show no phenotype for *bip-2* and *bip-3* as planarians can regenerate normally as in controls in either starved or feeding conditions. At the bottom are the number of planarians with the phenotype shown. (B) RNAi injections schedule in starved and feeding conditions for *bip-1* as performed for *cct3A* in Fig. 1. Live images show that RNAi for *bip-1* leads to no blastema formation compared to controls at 13dR during starved conditions. In feeding conditions at the same time point all planarians show anterior and posterior blastemas (black arrows), although some also show lysis at anterior blastemas (yellow arrows). By 44dR 40% *bip-1*(RNAi) planarians have regenerated. At the bottom are the number of planarians with the phenotype shown. The remaining planarians are dead at the indicated time points. All starved *bip-1(RNAi)* planarians were dead after 20dR. Scales, 500 μm.

A

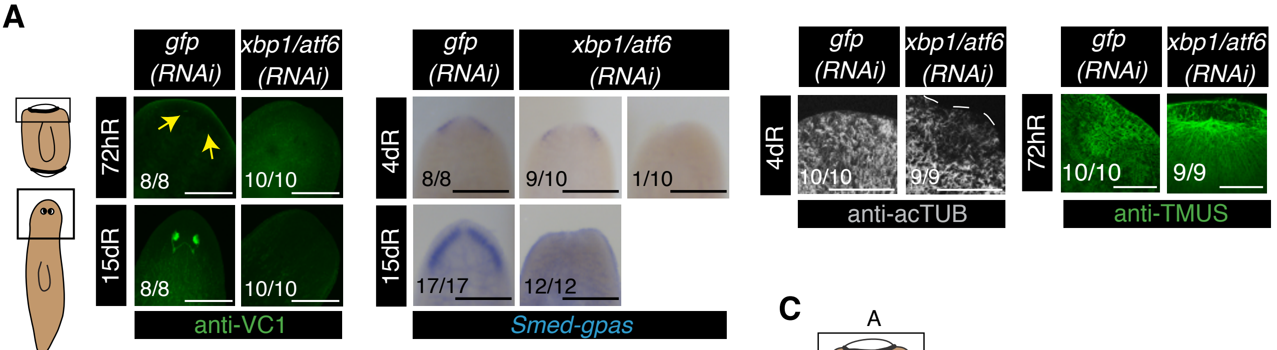

B

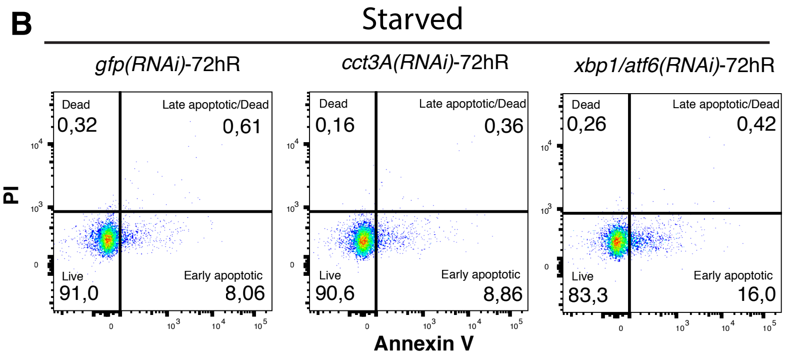

C

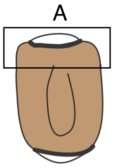

	Percentage of cells in different mitotic phases		Percentage of defective figures	
**72hR**	***gfp* (RNAi) n=42**	***xbp1/atf6* (RNAi); n=44**	***gfp* (RNAi)**	***xbp1/atf6* (RNAi)**
Prophase/ Prometaphase	19.05% (8/42)	27.27% (12/44)	0% (0/8)	41.67% (5/12)
Metaphase	42.86% (18/42)	25% (11/44)	0% (0/18)	54.55% (6/11)
Anaphase	38.10% (16/42)	47.73% (21/44)	0% (0/16)	66.67% (14/21)

Appendix Figure S4. *xbp1/atf6* RNAi phenocopies *cct3A* RNAi.
(A) The squares at the cartoons indicate the regions shown in the panels. The images show no differentiation of eyes (anti-VC1), and minimal differentiation of brain (*Smed-gpas*), epidermal cilia (anti-acTUB) and muscle (anti-TMUS) in anterior wounds of *xbp1/atf6* RNAi compared to controls in starved conditions. At the bottom are the number of planarians with the phenotype shown. Arrows indicate the differentiating eyes. (B) Representative plot showing living and dead X1 cells (stem cells) stained with propidium iodide (PI) and annexin V after *cct3A(RNAi)*, *xbp1/atf6(RNAi)* and controls in starved conditions at 72hR. Notice the increase in stem cell death after *xpb1/atf6(RNAi)* (sum of annexin V positive compartments: 16.42%) compared to controls (8.67%) or *cct3A(RNAi)* (9.92%). Approximately 7,000 X1 cells are displayed. $2x10^6$ total planarian cells were analyzed from 25 planarians per sample in two independent experiments. (C) Quantification of the percentage of stem cells in different mitotic phases and the percentage of defective mitotic figures in 72hR anterior blastemas of *xbp1/atf6* RNAi and controls during starvation after double immunostaining with anti-α-tubulin and anti-H3P. The cartoon displays the area analysed. A, anterior; hR, hours of regeneration; dR, days of regeneration. Scales, 300 µm (VC1 images), 500 µm (*gpas* images), 150 µm (TMUS and AC-TUB images).
