# Supplementary material for: Regeneration in starved planarians depends on TRiC/CCT subunits modulating the unfolded protein response
Source: EMBO Rep. 2021 Jun 30;22(8):e52905. doi: 10.15252/embr.202152905 (PMC8344900; doi:10.15252/embr.202152905)
Supplement: Supplementary file 2 — Expanded View Figures PDF [file EMBR-22-e52905-s005.pdf]

Expanded View Figures

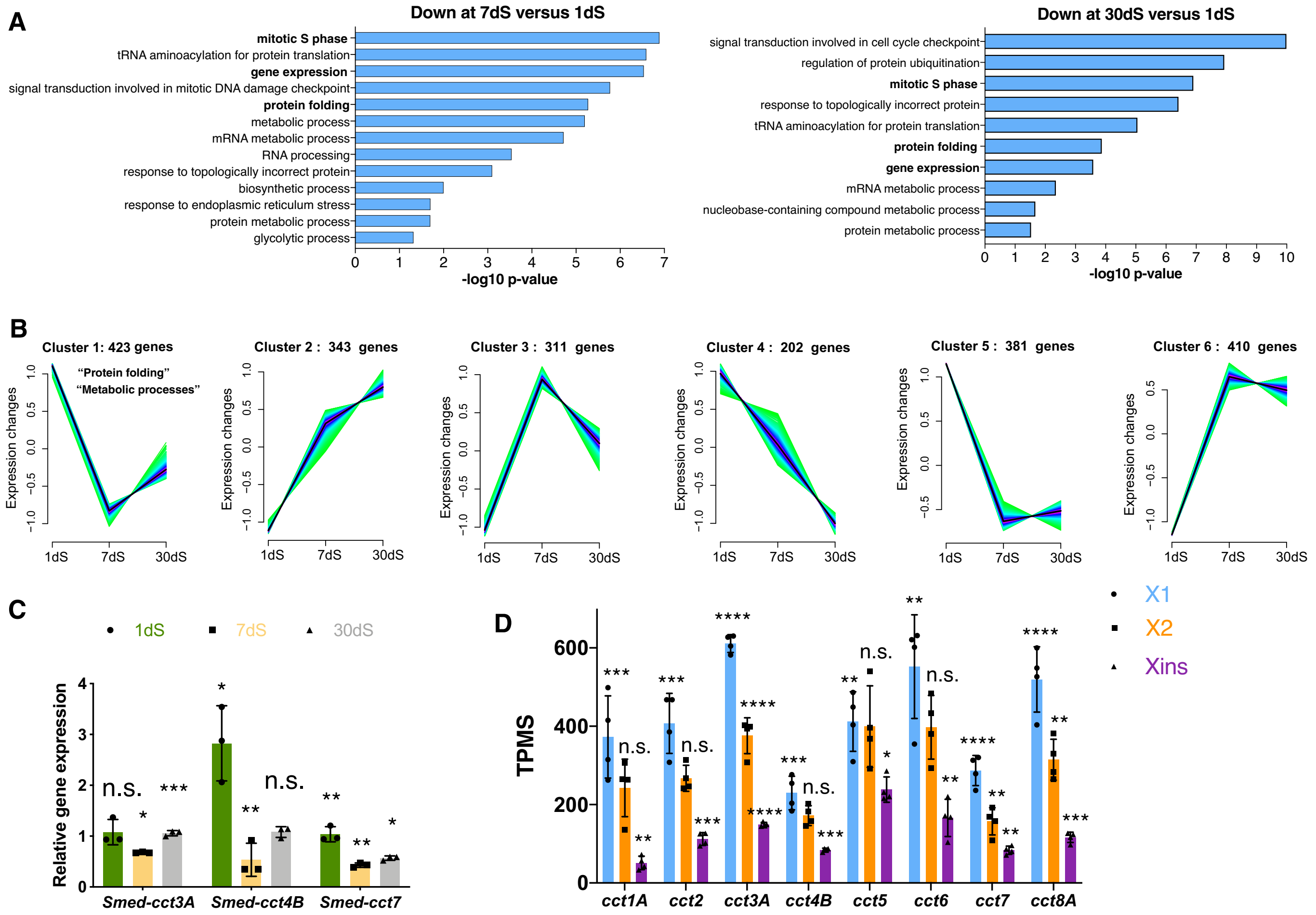

Figure EV1. Transcriptional profile of stem cells at different nutritional states finds TRiC subunits enriched in stem cells and differentially regulated at 1dS and 30dS versus 7dS.

A GO enrichment analysis for biological processes of the 814 down-regulated genes (q-value < 0.1) at 7dS versus 1dS and of the 491 down-regulated genes (q-value < 0.1) at 30dS versus 1dS in X1 (stem cells). q-value obtained by the Benjamini–Hochberg test. No enriched biological processes were found up-regulated. For better visualization, similar enriched GO terms based on the same subset of genes were manually removed to reduce redundancy. In bold are the GO terms commented in Results.

B Clustering generated from 2070 DEGs found in stem cells (X1) according to their expression during different nutritional conditions (1dS, 7dS and 30dS). Number of genes assigned to every cluster is showed on the upper part of every graph. Cluster 1 is characterized by a U shape.

C Relative expression of *cct* transcripts at 1, 7 and 30 days of starvation in X1 (stem cells). Error bars are SD from the mean. Asterisks refer to the condition just before and 1dS refers to 30dS and indicate $P < 0.001$ (three asterisks), $P < 0.01$ (two asterisks), $P < 0.05$ (one asterisk) and n.s. indicates not significant using two-tailed Student's test with equal sample variance. $n = 3$ replicates (5 planarians each) per time point.

D Expression levels of the different *cct* genes at 30 days of starvation in TPMs (transcripts per million). X2 corresponds to stem cell progeny (stem cells in G0/G1). These TMPs correspond to the data shown in Fig 1A. Error bars are SD from the mean. Asterisks refer to the condition before and X1 refers to Xins. $P < 0.0001$ (four asterisks), $P < 0.001$ (three asterisks), $P < 0.01$ (two asterisks), $P < 0.05$ (one asterisk) and n.s. indicates not significant using two-tailed Student's test with equal sample variance. $n = 4$ replicates (40 planarians per replicate).

Source data are available online for this figure.

© 2021 The Authors

Figure EV2. TRiC subunits are necessary for blastema formation specifically in starved planarians.

A Live images show that RNAi for any *cct* gene in starved conditions (14dS when the amputation is performed) leads to minimal blastema formation compared to controls at the time point shown whereas in feeding conditions (one extra feeding is introduced 1 day prior to injections) results in most planarians regenerating as controls. At the bottom are the number of planarians with the phenotype shown. The remaining planarians are dead by the time point of regeneration shown.

B RNAi injections schedule in starved conditions (37 days of starvation when the amputation is performed which is indicated by the grey cross at day 37). Live images show that *cct3A(RNAi)* planarians form a minimal blastema when compared to controls at the time points shown. At the bottom are the number of planarians with the phenotype shown.

C Expression of *cct3A* in *cct3A(RNAi)* planarians related to *gfp* RNAi injected either under starved (14dS) or feeding conditions at 1 and 13 days of regeneration. Error bars are SD from the mean. $P < 0.0001$ (four asterisks), $P < 0.001$ (three asterisks) and n.s. indicates not significant using two-tailed Student's test with equal sample variance. $n = 3$ replicates (5 planarians each) per time point.

Data information: dR, days of regeneration. Scale bars, 500 μm.

Source data are available online for this figure.

 © 2021 The Authors

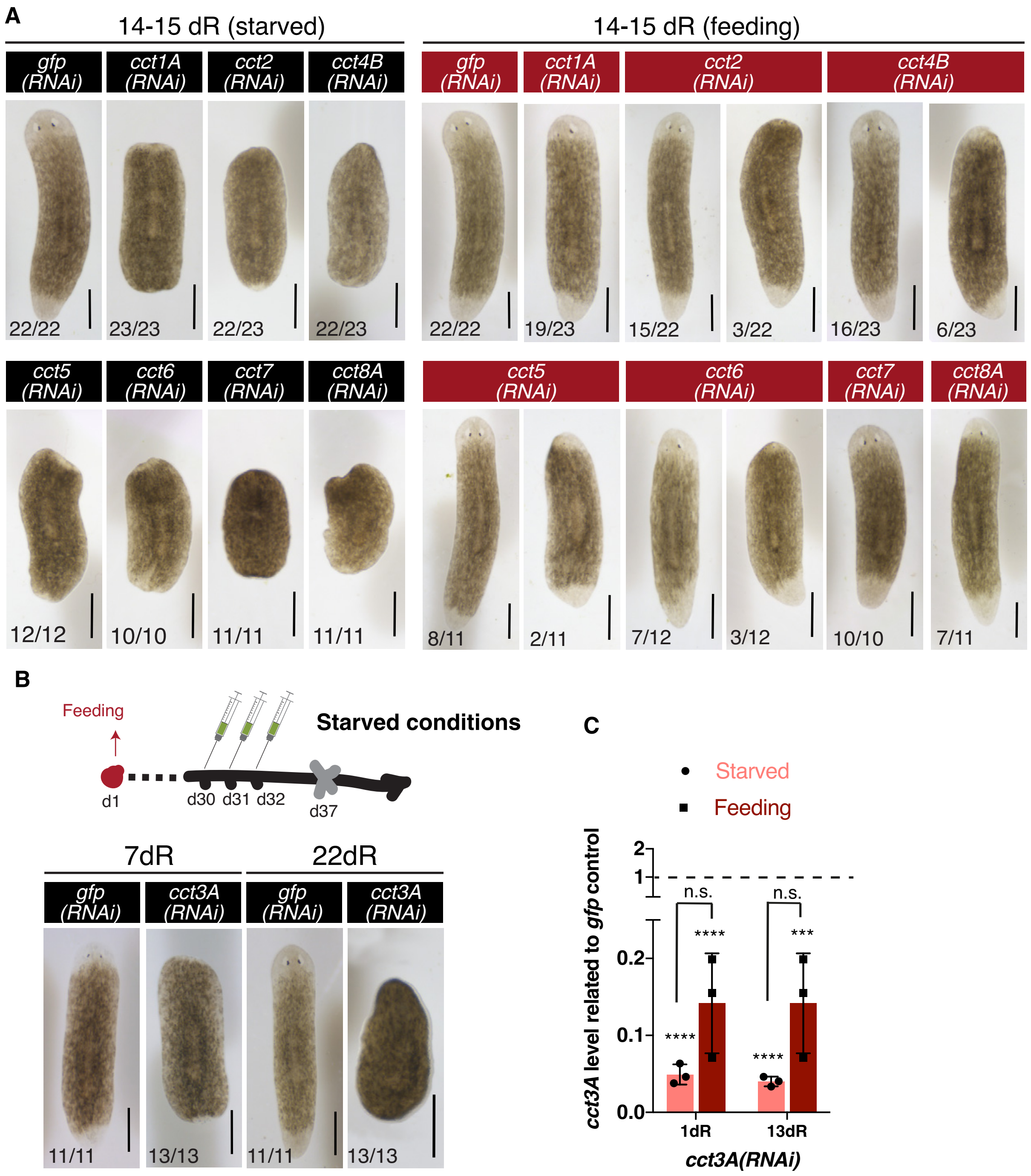
A
14-15 dR (starved)
14-15 dR (feeding)
gfp (RNAi)
cct1A (RNAi)
cct2 (RNAi)
cct4B (RNAi)
22/22
23/23
22/23
22/23
gfp (RNAi)
cct1A (RNAi)
cct2 (RNAi)
cct4B (RNAi)
22/22
19/23
15/22
3/22
16/23
6/23
cct5 (RNAi)
cct6 (RNAi)
cct7 (RNAi)
cct8A (RNAi)
12/12
10/10
11/11
11/11
cct5 (RNAi)
cct6 (RNAi)
cct7 (RNAi)
cct8A (RNAi)
8/11
2/11
7/12
3/12
10/10
7/11
B
Feeding
Starved conditions
d1
d30 d31 d32
d37
7dR
22dR
gfp (RNAi)
cct3A (RNAi)
gfp (RNAi)
cct3A (RNAi)
11/11
13/13
11/11
13/13
C
Starved
Feeding
cct3A level related to gfp control
n.s.
n.s.
****
***
****
****
1dR
13dR
cct3A(RNAi)

Figure EV2.

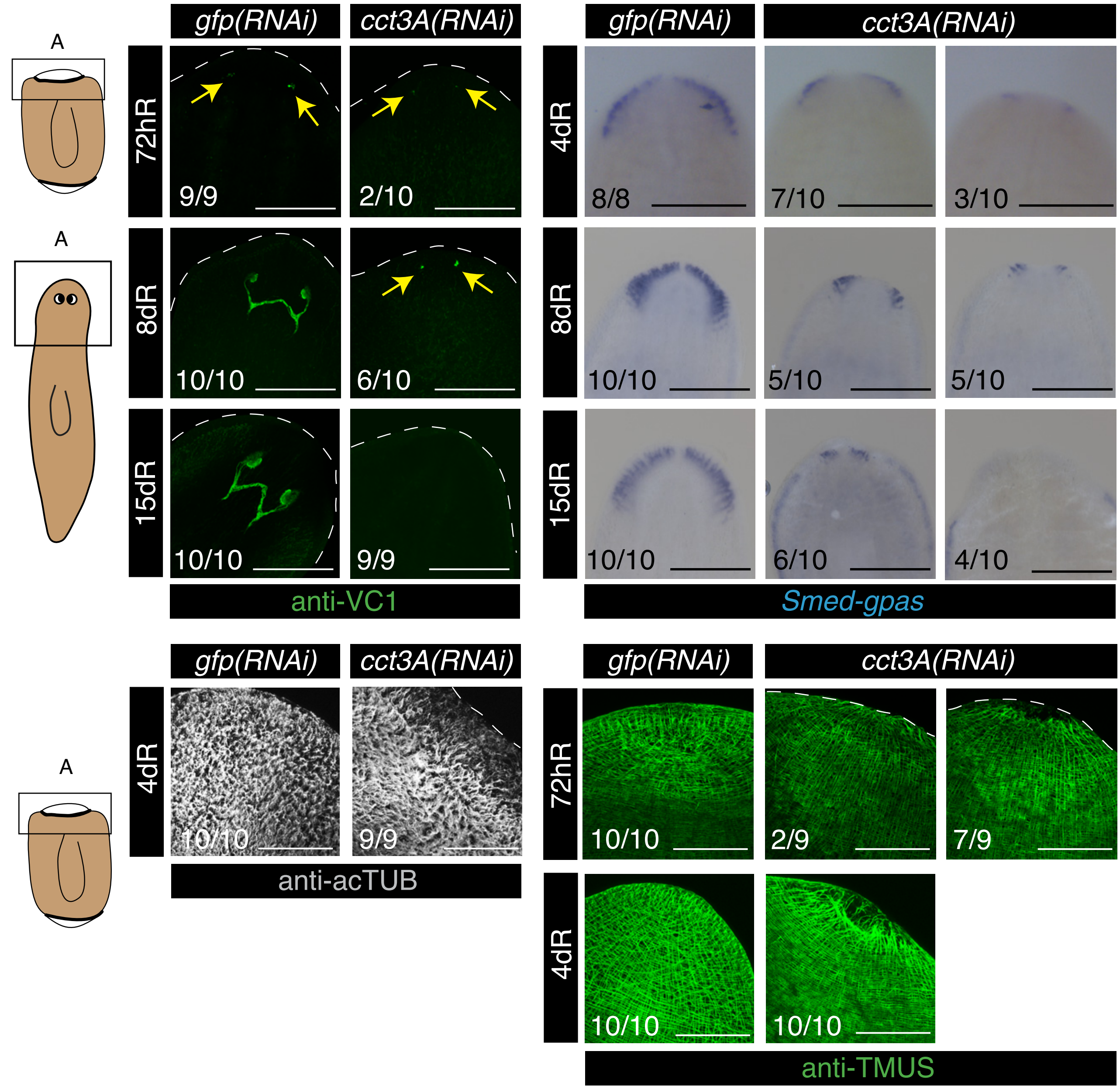

Figure EV3. ***cct3A*** **RNAi show minimal differentiation during regeneration in starved conditions.**

The squares at the cartoons indicate the regions shown in the panels. The images show minimal differentiation of eyes (anti-VC1), brain (*Smed-gpas*), epidermal cilia (anti-acTUB) and muscle (anti-TMUS) in anterior wounds of *cct3A* RNAi compared to controls. At the bottom are the number of planarians with the phenotype shown. Arrows indicate the differentiating eyes. Scale bars, 300 µm (VC1 images), 500 µm (*gpas* images), 150 µm (TMUS and AC-TUB images). hR indicates hours of regeneration, and dR indicates days of regeneration.

Source data are available online for this figure.

 © 2021 The Authors

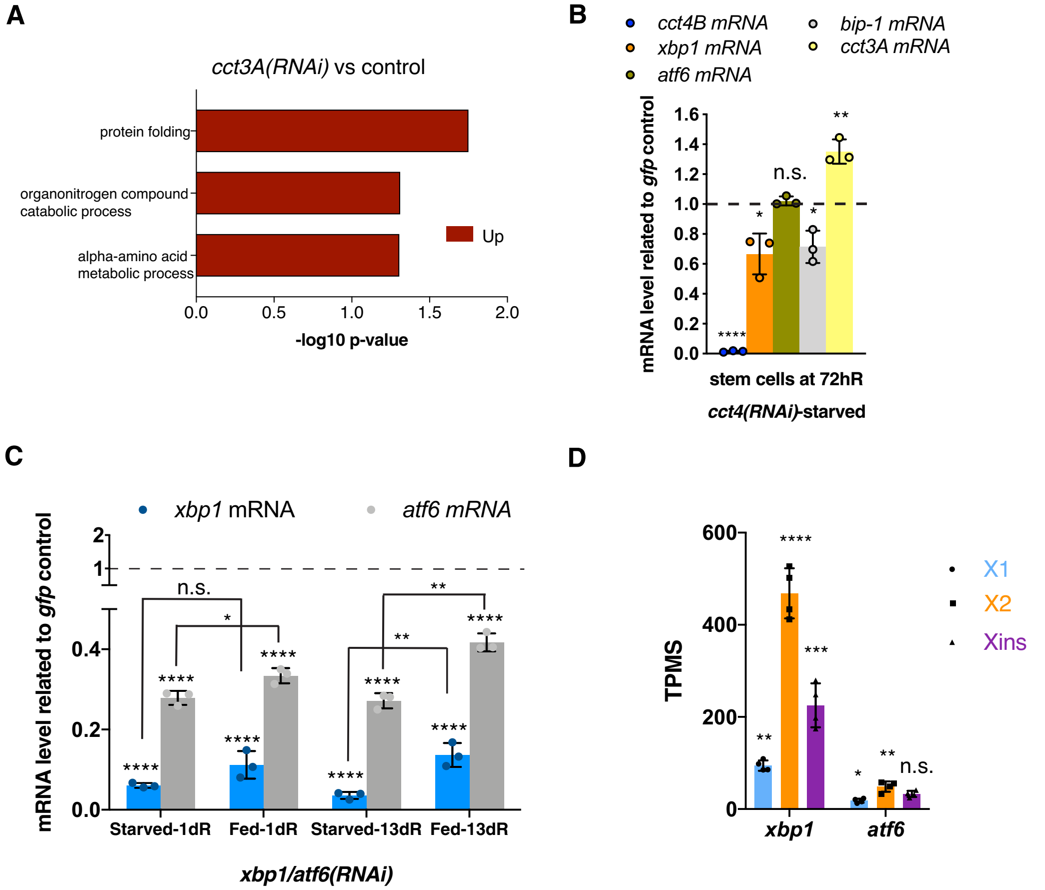

Figure EV4. *cct3A* down-regulates components of the UPR.

A GO enrichment analysis for biological processes of the 982 up-regulated genes (q-value < 0.05) in starved *cct3A* RNAi planarians versus controls at 72hR. q-value was obtained with the Benjamini–Hochberg test. No enriched biological processes were found down-regulated. For better visualization, similar enriched GO terms based on the same subset of genes were manually removed to reduce redundancy.

B The graph shows that *cct4B* RNAi down-regulates *xbp1* and *bip-1* and up-regulates *cct3A* specifically at 72hR during starvation in stem cells. Error bars are SD from the mean. 50 planarians per replicate (3 replicates) were used to obtain the X1 population (stem cells). $P < 0.0001$ (four asterisks), $P < 0.01$ (two asterisks), $P < 0.05$ (one asterisk) and n.s. indicates not significant using two-tailed Student's test with equal sample variance.

C Expression of *xbp1 and atf6* at 1 and 13 days of regeneration after *xbp1/atf6(RNAi)* related to controls during either starving or feeding conditions. The graphs show that *xbp1/atf6* RNAi down-regulates *xbp1* and *atf6* during starvation and feeding at both time points. Error bars are SD from the mean. Asterisks indicate $P < 0.0001$ (four asterisks), $P < 0.01$ (two asterisks) and $P < 0.05$ (one asterisk), and n.s. indicates not significant using two-tailed Student's test with equal sample variance. $n = 3$ replicates (5 planarians each) per time point.

D Expression levels of *xbp1 and atf6* at 30 days of starvation in the X1, X2 and Xins FACS populations in TPMs (transcripts per million). Error bars are SD from the mean. Asterisks refer to the condition before and X1 refers to Xins and indicate $P < 0.0001$ (four asterisks), $P < 0.001$ (three asterisks), $P < 0.01$ (two asterisks) and $P < 0.05$ (one asterisk), and n.s. indicates not significant using two-tailed Student's test with equal sample variance. $n = 4$ replicates (40 planarians each) per time point.

Source data are available online for this figure.

Figure EV5. Supplementary experiments on DTT treatments.

A Expression of *bip-1* in 72hR planarians in starved conditions related to 72hR planarians in feeding conditions. Error bars are SD from the mean. $P < 0.01$ (two asterisks) using two-tailed Student's test with equal sample variance. $n = 3$ replicates (5 planarians each).

B, C The Kaplan–Meier curve shows 100% survival of intact planarians treated with 0.05 mM DTT in both starved and feeding conditions. 0.2 mM and 0.1 mM DTT leads to death of all treated animals in feeding conditions by day 14. While 0.2 mM DTT in starved planarians leads to some death, the rest of the planarians showed wounds and lysis by day 14 as displayed in the images. All starved planarians treated with 0.1 mM DTT were normal, and after amputation at day 14, they all regenerated normally. At the concentration of 0.05 mM all planarians, both in the starved and feeding conditions, looked normal after the treatments and regenerated well after the amputation. Four asterisks indicate $P < 0.0001$ of 0.2 mM DTT and 0.1 mM DTT treatments on the feeding conditions respect to controls, and n.s. indicates not significant respect to controls with log-rank (Mantel–Cox) test. The rest of conditions are not significant. Images are representative surviving animals for the different conditions. At the bottom are the number of planarians with the phenotype shown from a total of 31 planarians per condition in two independent experiments. The rest of planarians are dead at this time point. Scale bars, 1 mm.

D Expression of *bip-1* in planarians in starved and feeding conditions 1 h after the last day of treatment with DTT (0.05 and 0.2 mM). The levels of *bip-1* increase after exposure to DTT and the increase is bigger with higher doses of DTT. Error bars are SD from the mean. $P < 0.0001$ (four asterisks), $P < 0.01$ (two asterisks) and $P < 0.05$ (one asterisk) using two-tailed Student's test with equal sample variance. $n = 3$ replicates (5 planarians each) per time point.

E The Kaplan–Meier curve demonstrates decreased survival of *cct3A* RNAi animals when treated with DTT under feeding conditions. Percentages indicate the number of survivals at the indicated time points. Two asterisks indicate $P < 0.01$ with log-rank (Mantel–Cox) test. Images are representative surviving animals for the different conditions at this time point. At the bottom are the number of planarians with the phenotype shown. The rest of planarians are dead at the displayed time point. dR indicates days of regeneration. Scale bars, 500 μm.

Source data are available online for this figure.

 © 2021 The Authors

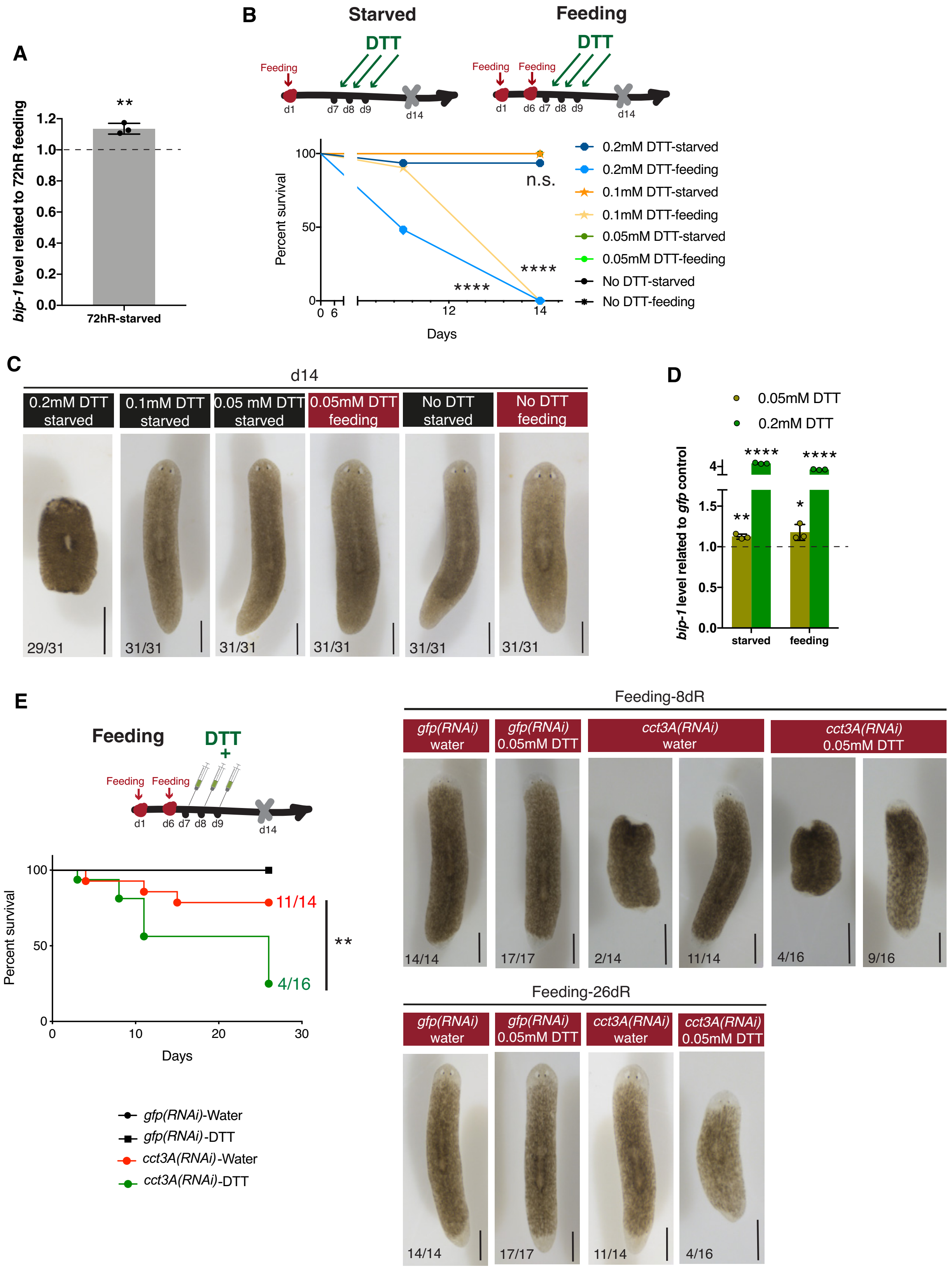

A
B
Starved
Feeding
DTT
Feeding
d1 d7 d8 d9 d14
d1 d6 d7 d8 d9 d14
bip-1 level related to 72hR feeding
72hR-starved
Percent survival
Days
n.s.
****
0.2mM DTT-starved
0.2mM DTT-feeding
0.1mM DTT-starved
0.1mM DTT-feeding
0.05mM DTT-starved
0.05mM DTT-feeding
No DTT-starved
No DTT-feeding
C
d14
0.2mM DTT starved
0.1mM DTT starved
0.05 mM DTT starved
0.05mM DTT feeding
No DTT starved
No DTT feeding
29/31
31/31
D
0.05mM DTT
0.2mM DTT
bip-1 level related to gfp control
starved
feeding
E
Feeding
DTT
Feeding-8dR
Feeding-26dR
gfp(RNAi) water
gfp(RNAi) 0.05mM DTT
cct3A(RNAi) water
cct3A(RNAi) 0.05mM DTT
14/14
17/17
2/14
11/14
4/16
9/16
gfp(RNAi)-Water
gfp(RNAi)-DTT
cct3A(RNAi)-Water
cct3A(RNAi)-DTT

Figure EV5.
