# Supplementary material for: Regeneration in starved planarians depends on TRiC/CCT subunits modulating the unfolded protein response
Source: EMBO Rep. 2021 Jun 30;22(8):e52905. doi: 10.15252/embr.202152905 (PMC8344900; doi:10.15252/embr.202152905)
Supplement: Supplementary file 8 — Review Process File [file EMBR-22-e52905-s013.pdf]

EMBO reports

Regeneration in starved planarians depends on TRiC/CCT subunits modulating the unfolded protein response

Óscar Gutiérrez-Gutiérrez, Daniel Felix, Alessandra Salvetti, Elias Amro, Anne Thems, Stefan Pietsch, Andreas Koeberle, Karl-Lenhard Rudolph and Cristina González-Estévez
DOI: 10.15252/embr.202152905

Corresponding author(s): Cristina González-Estévez (cristina.gonzalez.estevez@gmail.com)

Review Timeline:

Submission Date:	30th Mar 21
Editorial Decision:	22nd Apr 21
Revision Received:	6th May 21
Editorial Decision:	12th May 21
Revision Received:	15th May 21
Accepted:	20th May 21

Editor: Esther Schnapp

Transaction Report:

Please note that the manuscript was previously reviewed at another journal and the reports were taken into account in the decision making process at EMBO Reports. Since the original reviews are not subject to EMBO Press' transparent review process policy, the reports and author response cannot be published.

(Note: With the exception of the correction of typographical or spelling errors that could be a source of ambiguity, letters and reports are not edited. Depending on transfer agreements, referee reports obtained elsewhere may or may not be included in this compilation. Referee reports are anonymous unless the Referee chooses to sign their reports.)

EMBO PRESS

YOU MUST COMPLETE ALL CELLS WITH A PINK BACKGROUND ↓

PLEASE NOTE THAT THIS CHECKLIST WILL BE PUBLISHED ALONGSIDE YOUR PAPER

Corresponding Author Name: Cristina González-Estévez
Journal Submitted to: EMBO reports
Manuscript Number: EMBOR-2021-52905V3

Reporting Checklist For Life Sciences Articles (Rev. June 2017)

This checklist is used to ensure good reporting standards and to improve the reproducibility of published results. These guidelines are consistent with the Principles and Guidelines for Reporting Preclinical Research issued by the NIH in 2014. Please follow the journal's authorship guidelines in preparing your manuscript.

A- Figures
1. Data
The data shown in figures should satisfy the following conditions:

- ➔ the data were obtained and processed according to the field's best practice and are presented to reflect the results of the experiments in an accurate and unbiased manner.
- ➔ figure panels include only data points, measurements or observations that can be compared to each other in a scientifically meaningful way.
- ➔ graphs include clearly labeled error bars for independent experiments and sample sizes. Unless justified, error bars should not be shown for technical replicates.
- ➔ if n< 5, the individual data points from each experiment should be plotted and any statistical test employed should be justified
- ➔ Source Data should be included to report the data underlying graphs. Please follow the guidelines set out in the author ship guidelines on Data Presentation.

2. Captions
Each figure caption should contain the following information, for each panel where they are relevant:

- ➔ a specification of the experimental system investigated (eg cell line, species name).
- ➔ the assay(s) and method(s) used to carry out the reported observations and measurements
- ➔ an explicit mention of the biological and chemical entity(ies) that are being measured.
- ➔ an explicit mention of the biological and chemical entity(ies) that are altered/varied/perturbed in a controlled manner.

- ➔ the exact sample size (n) for each experimental group/condition, given as a number, not a range;
- ➔ a description of the sample collection allowing the reader to understand whether the samples represent technical or biological replicates (including how many animals, litters, cultures, etc.).
- ➔ a statement of how many times the experiment shown was independently replicated in the laboratory.
- ➔ definitions of statistical methods and measures:
  - common tests, such as t-test (please specify whether paired vs. unpaired), simple $\chi 2$ tests, Wilcoxon and Mann-Whitney tests, can be unambiguously identified by name only, but more complex techniques should be described in the methods section;
  - are tests one-sided or two-sided?
  - are there adjustments for multiple comparisons?
  - exact statistical test results, e.g., P values = x but not P values < x;
  - definition of 'center values' as median or average;
  - definition of error bars as s.d. or s.e.m.

Any descriptions too long for the figure legend should be included in the methods section and/or with the source data.

In the pink boxes below, please ensure that the answers to the following questions are reported in the manuscript itself. Every question should be answered. If the question is not relevant to your research, please write NA (non applicable). We encourage you to include a specific subsection in the methods section for statistics, reagents, animal models and human subjects.

USEFUL LINKS FOR COMPLETING THIS FORM

http://www.antibodypedia.com
http://1degreebio.org
http://www.equator-network.org/reporting-guidelines/improving-bioscience-research-repor
http://grants.nih.gov/grants/olaw/olaw.htm
http://www.mrc.ac.uk/Ourresearch/Ethicsresearchguidance/Useofanimals/index.htm
http://ClinicalTrials.gov
http://www.consort-statement.org
http://www.consort-statement.org/checklists/view/32-consort/66-title
http://www.equator-network.org/reporting-guidelines/reporting-recommendations-for-tumo
http://datadryad.org
http://figshare.com
http://www.ncbi.nlm.nih.gov/gap
http://www.ebi.ac.uk/ega
http://biomodels.net/
http://biomodels.net/miriam/
http://jjj.biochem.sun.ac.za
http://oba.od.nih.gov/biosecurity/biosecurity_documents.html
http://www.selectagents.gov/

B- Statistics and general methods

Please fill out these boxes ↓ (Do not worry if you cannot see all your text once you press return)

1.a. How was the sample size chosen to ensure adequate power to detect a pre-specified effect size?	No sample size calculations were performed. Sample sizes were chosen to be similar to previously published data for highly penetrant phenotypes.
1.b. For animal studies, include a statement about sample size estimate even if no statistical methods were used.	At least 5 planarians per independent experiment. In case of qPCR we always did 3 biological replicates with 5 planarians per replicate. At least 3 mice were used per experiment.
2. Describe inclusion/exclusion criteria if samples or animals were excluded from the analysis. Were the criteria pre-established?	No data was excluded from analyses. The only exception was if a planarian was completly broken because of handling during FISH or ISH.
3. Were any steps taken to minimize the effects of subjective bias when allocating animals/samples to treatment (e.g. randomization procedure)? If yes, please describe.	Animals were randomly assigned to RNAi condition and stainings
For animal studies, include a statement about randomization even if no randomization was used.	Animals were randomly assigned to RNAi condition and stainings
4.a. Were any steps taken to minimize the effects of subjective bias during group allocation or/and when assessing results (e.g. blinding of the investigator)? If yes please describe.	Investigators were not blinded during data acquisition or analysis
4.b. For animal studies, include a statement about blinding even if no blinding was done	Investigators were not blinded during data acquisition or analysis
5. For every figure, are statistical tests justified as appropriate?	Yes
Do the data meet the assumptions of the tests (e.g., normal distribution)? Describe any methods used to assess it.	Yes. When appropiate we peformed D'Agostino and Pearson normality test
Is there an estimate of variation within each group of data?	The tests that we performed assumed equal sample variance

Is the variance similar between the groups that are being statistically compared?	Yes

C- Reagents

6. To show that antibodies were profiled for use in the system under study (assay and species), provide a citation, catalog number and/or clone number, supplementary information or reference to an antibody validation profile. e.g., Antibodypedia (see link list at top right), 1DegreeBio (see link list at top right).	anti-VC-1 (diluted 1/15000; kindly provided by Professor K. Watanabe and H. Orii); anti-TMUS13 (diluted 1/20; kindly provided by Professor R. Romero); anti-acetylated-tubulin (diluted 1:200, Sigma; clone 6-11B-1); and anti-Histone H3 phosphorylated at serine 10 (H3P, diluted 1/500, Santa Cruz; sc-8656-R); anti-Tubulin Tyrosin (diluted 1/400; Sigma; T9028); anti–c-Kit-APC (Biolegend; 105812); CD4 (Biolegend; 100508), CD8 (Biolegend; 100704), TER-119 (Biolegend; 116204), CD11b (Biolegend; 101204), Gr-1 (Biolegend; 108404), and B220 (Biolegend; 103222); anti–Sca-1-PE-Cy7 (Biolegend; 108114); streptavidin-APC-Cy7 (Biolegend; 405208)
7. Identify the source of cell lines and report if they were recently authenticated (e.g., by STR profiling) and tested for mycoplasma contamination.	Does no apply

* for all hyperlinks, please see the table at the top right of the document

D- Animal Models

8. Report species, strain, gender, age of animals and genetic modification status where applicable. Please detail housing and husbandry conditions and the source of animals.	Asexual planarians were used for all experiments and maintained under standard conditions; C57BL/6J wild-type mice (5-6-month-old).
9. For experiments involving live vertebrates, include a statement of compliance with ethical regulations and identify the committee(s) approving the experiments.	Mice used in this study were C57BL/6J wild-type mice (5-6-month-old) obtained from Janvier or from internal stock (FLI internal license O_KLR_18-20 and §11 Lizenz). Mice were maintained in a specific pathogen-free animal facility in Fritz Lipmann Institute with 12 h of light/dark cycle and fed with a standard mouse chow. Animal experiments were performed according to Institutional and European Union guidelines.
10. We recommend consulting the ARRIVE guidelines (see link list at top right) (PLoS Biol. 8(6), e1000412, 2010) to ensure that other relevant aspects of animal studies are adequately reported. See author guidelines, under 'Reporting Guidelines'. See also: NIH (see link list at top right) and MRC (see link list at top right) recommendations. Please confirm compliance.	We confirm compliance.

E- Human Subjects

11. Identify the committee(s) approving the study protocol.	Does not apply
12. Include a statement confirming that informed consent was obtained from all subjects and that the experiments conformed to the principles set out in the WMA Declaration of Helsinki and the Department of Health and Human Services Belmont Report.	Does not apply
13. For publication of patient photos, include a statement confirming that consent to publish was obtained.	Does not apply
14. Report any restrictions on the availability (and/or on the use) of human data or samples.	Does not apply
15. Report the clinical trial registration number (at ClinicalTrials.gov or equivalent), where applicable.	Does not apply
16. For phase II and III randomized controlled trials, please refer to the CONSORT flow diagram (see link list at top right) and submit the CONSORT checklist (see link list at top right) with your submission. See author guidelines, under 'Reporting Guidelines'. Please confirm you have submitted this list.	Does not apply
17. For tumor marker prognostic studies, we recommend that you follow the REMARK reporting guidelines (see link list at top right). See author guidelines, under 'Reporting Guidelines'. Please confirm you have followed these guidelines.	Does not apply

F- Data Accessibility

18: Provide a "Data Availability" section at the end of the Materials & Methods, listing the accession codes for data generated in this study and deposited in a public database (e.g. RNA-Seq data: Gene Expression Omnibus GSE39462, Proteomics data: PRIDE PXD000208 etc.) Please refer to our author guidelines for 'Data Deposition'.<br><br>Data deposition in a public repository is mandatory for:<br>a. Protein, DNA and RNA sequences<br>b. Macromolecular structures<br>c. Crystallographic data for small molecules<br>d. Functional genomics data<br>e. Proteomics and molecular interactions	Sequence of cct8A has been previously deposited in GenBank: MF669570 (Counts et al., 2017). Sequences of xbp1, atf6, cct3A, cct1A, cct2, cct4B, cct5, cct7, cct6, bip-1, bip-2 and bip-3 have been deposited in GenBank with the accession numbers MN171093-MN171100, MN380639 and MT411562-MT411564. RNA-Seq data has been deposited in GEO with the accession numbers GSE134148 and GSE134013
19. Deposition is strongly recommended for any datasets that are central and integral to the study; please consider the journal's data policy. If no structured public repository exists for a given data type, we encourage the provision of datasets in the manuscript as a Supplementary Document (see author guidelines under 'Expanded View' or in unstructured repositories such as Dryad (see link list at top right) or Figshare (see link list at top right).	All datasets and source data for all figures has been submitted.
20. Access to human clinical and genomic datasets should be provided with as few restrictions as possible while respecting ethical obligations to the patients and relevant medical and legal issues. If practically possible and compatible with the individual consent agreement used in the study, such data should be deposited in one of the major public access-controlled repositories such as dbGAP (see link list at top right) or EGA (see link list at top right).	Does not apply.
21. Computational models that are central and integral to a study should be shared without restrictions and provided in a machine-readable form. The relevant accession numbers or links should be provided. When possible, standardized format (SBML, CellML) should be used instead of scripts (e.g. MATLAB). Authors are strongly encouraged to follow the MIRIAM guidelines (see link list at top right) and deposit their model in a public database such as Biomodels (see link list at top right) or JWS Online (see link list at top right). If computer source code is provided with the paper, it should be deposited in a public repository or included in supplementary information.	Does not apply.

G- Dual use research of concern

22. Could your study fall under dual use research restrictions? Please check biosecurity documents (see link list at top right) and list of select agents and toxins (APHIS/CDC) (see link list at top right). According to our biosecurity guidelines, provide a statement only if it could.	Does not apply.
